# Radiation dose rate effects: what is new and what is needed?

**DOI:** 10.1007/s00411-022-00996-0

**Published:** 2022-10-15

**Authors:** Donna Lowe, Laurence Roy, Maria Antonella Tabocchini, Werner Rühm, Richard Wakeford, Gayle E. Woloschak, Dominique Laurier

**Affiliations:** 1 UK Health Security Agency, CRCE Chilton, Didcot, OX11 0RQ Oxfordshire UK; 2 Institut de Radioprotection Et de Sûreté Nucléaire, Fontenay-Aux-Roses, France; 3 Istituto Nazionale i Fisica Nucleare, Sezione i Roma, Rome, Italy; 4 Istituto Superiore Di Sanità, Rome, Italy; 5 Institute of Radiation Medicine, Helmholtz Center Munich, Ingolstädter Landstr. 1, 85764 Neuherberg, Germany; 6 Centre for Occupational and Environmental Health, The University of Manchester, Manchester, M13 9PL UK; 7 Department of Radiation Oncology, Northwestern University School of Medicine, Chicago, IL USA

**Keywords:** Ionising radiation, Dose rate, Low-LET, Recommendations, Radiobiology, Epidemiology

## Abstract

Despite decades of research to understand the biological effects of ionising radiation, there is still much uncertainty over the role of dose rate. Motivated by a virtual workshop on the “Effects of spatial and temporal variation in dose delivery” organised in November 2020 by the Multidisciplinary Low Dose Initiative (MELODI), here, we review studies to date exploring dose rate effects, highlighting significant findings, recent advances and to provide perspective and recommendations for requirements and direction of future work. A comprehensive range of studies is considered, including molecular, cellular, animal, and human studies, with a focus on low linear-energy-transfer radiation exposure. Limits and advantages of each type of study are discussed, and a focus is made on future research needs.

## Introduction

In the current system of radiological protection, risk to a specific organ or tissue is considered to depend on the absorbed energy averaged over the target mass exposed. The biological outcome of the exposure is determined not only by the total absorbed dose but also by the time frame of the dose delivery, and by the type of ionising radiation responsible for the energy deposition (radiation quality). To account for the effects of dose and the temporal variation in dose delivery, a single dose and dose rate effectiveness factor (DDREF) is currently applied for the purposes of radiological protection. However, the evidence base for this judgement continues to be debated, as reflected by previous and ongoing work performed in Task Group 91 of the International Commission on Radiological Protection (ICRP) (Rühm et al. 2015, 2016; Wakeford et al. 2019).

The EU MELODI (Multidisciplinary Low Dose Initiative) platform is considering inhomogeneity in dose delivery, both at the temporal and spatial level, as a priority research area. Mechanisms responsible for biological effects of different dose rates or of inhomogeneous spatial dose deposition are not fully characterised. At the cellular level, such effects are investigated with in vitro studies, but when it comes to how they finally affect human health risk (both cancer and non-cancer diseases), few relevant experimental models or validated datasets exist (https://melodi-online.eu/). To cover the topic of the effects of spatial and temporal variation in dose delivery, a digital workshop was conducted in November 2020 evaluating what is known on the effect of dose rate, among other aspects. This publication builds on the outcomes of this meeting.

The present paper summarises current evidence for the influence of dose rate upon radiation-related effects. Endpoints considered include molecular, cellular, organism, and human studies. Emphasis will be placed on dose rates relevant for radiological protection settings. We focus on low linear-energy-transfer (LET) external exposures, since for internal contamination with radionuclides, a decrease in dose rate with time will occur to varying extents due to the physical and biological half-lives of the involved radionuclides, complicating the interpretation of results.

The manuscript structure includes the history of low-dose rate definition, ongoing work on DDREF under ICRP TG91, presentations of experimental work (in vitro and in vivo), and epidemiological studies. Limits and advantages of each approach are discussed, and a focus is made on future research needs.

## The dose rate concept

### Definition of low-dose rate

The United Nations Scientific Committee on the Effects of Atomic Radiation (UNSCEAR) first defined low-dose rate (LDR) with respect to radiation-related cancer in its 1986 Report (UNSCEAR 1986). For all types of radiation, LDR were < 0.05 mGy/min (3 mGy/h) and high-dose rate (HDR) were > 0.05 Gy/min (3000 mGy/h), with dose rates in between defined as “intermediate dose rates”. These definitions were reiterated in the UNSCEAR (1988) Report. The UNSCEAR (1993) Report, Annex F comprehensively discussed how dose rates might be classified according to a number of approaches: microdosimetric considerations, cellular experiments, animal experiments, and human epidemiology. UNSCEAR (1993) concluded that information on LDR relevant to assessing radiation carcinogenesis in humans could be obtained from animal experiments. On the basis of animal studies, UNSCEAR (1993) was of the view that following exposure to low-LET radiation, a dose rate effectiveness factor should be applied to reduce the excess cancer risk per unit dose if the dose rate was < 0.1 mGy/min (when averaged over about an hour), whatever the total dose received.

Of interest is the position adopted by ICRP in Publication 60, the 1990 Recommendations (ICRP 1991). In ICRP Publication 60, in the context of stochastic health effects, an LDR was defined as < 0.1 Gy/h (equivalent to 1.67 mGy/min), a dose rate that was a factor of 33 larger than the < 0.05 mGy/min defined by UNSCEAR in its 1988 Report, but no explanation was provided as to how this value was derived or why it differed substantially from the definition then recently adopted by UNSCEAR (1988). The ICRP Publication 60 definition of an LDR as < 0.1 Gy/h contrasts with that of < 0.1 Gy/day adopted by the UK National Radiological Protection Board (NRPB) in 1988 in its report NRPB-R226 (Stather et al. 1988) and referred to in the UNSCEAR 1993 Report (UNSCEAR 1993). A definition of an LDR as < 0.1 Gy/day is equivalent to < 0.07 mGy/min, which is very close to the definition given in the UNSCEAR 1988 Report of < 0.05 mGy/min. The ICRP 2007 Recommendations, ICRP Publication 103 (ICRP 2007), although frequently referring to LDR in the context of a low-dose rate effectiveness factor, does not define the range of dose rates considered to be LDR. However, the recently published ICRP Publication 147 (Harrison et al. 2021a, 2021b) states that a DDREF should not be applied to reduce solid cancer risks if the dose rate for low-LET radiation exceeds 5 mGy/h, implying a definition of LDR of 0.1 mGy/min when averaged over approximately 1 h, which is the definition of LDR as restated in the UNSCEAR 2019 Report (UNSCEAR 2019)   and in the 2020/2021 Report (UNSCEAR 2021) defines a low dose rate for hight-LET radiation as "no more than one high-LET track traversal per cell per hour".

The above definitions of LDR have been established with respect to stochastic effects, especially cancers. We note, however, that ICRP Publication 60 mentions in the context of deterministic effects that dose rates lower than 0.1 Gy/min of low-LET radiation “result in progressively less cell killing until a dose rate of about 0.1 Gy/h or less is reached for mammalian cells” (ICRP 1991).

### Low-dose rate in the current system of radiological protection

In the current scheme of radiological protection recommended by ICRP, following the definitions used by UNSCEAR, for an exposure to a low dose (conventionally < 100 mGy of low-LET radiation) or for an exposure at an LDR (< 0.1 mGy/min of low-LET radiation when averaged over about 1 h, i.e., approximately 5 mGy/h), the excess risk of adverse stochastic health effects (cancer in the exposed individual and hereditary disease in the subsequently conceived descendants of the exposed individual) is taken to be directly proportional to the dose of radiation received with no-threshold dose below which there is an absence of excess risk. This is the linear no-threshold (LNT) dose–response model.

For low-level exposures (low doses or LDR), the current ICRP recommendations incorporate a DDREF, which reduces the risk per unit dose when risk estimates derived from exposures to moderate-to-high doses received at an HDR are applied to exposures to low doses or LDR. Risk estimates for solid cancers obtained from the Japanese atomic-bomb survivors are halved (corresponding to a DDREF of 2) when applied to low-level exposures. A DDREF is not applied to leukaemia, because a linear-quadratic dose–response model is used (rather than linear dose–response models used for solid cancers), which is implicitly consistent with a reduction of risk at low levels of exposure (Cléro et al. 2019).

The DDREF can be considered a combination of a low-dose effectiveness factor (LDEF) and a dose rate effectiveness factor (DREF). The LDEF essentially addresses the degree of upward curvature of the dose–response following a range of doses received from acute exposures to low-LET radiation, whereas the DREF compares the risk per unit dose following high and LDR exposures. Here, epidemiological evidence will be examined to assess the degree of support for the application of a DREF (and specifically, a DREF of 2) to the risk per unit dose obtained from the Japanese atomic-bomb survivors to obtain the risk per unit dose appropriate for LDR exposures.

### Recent positions on DDREF

The numerical value of the DDREF is internationally debated. ICRP, in its Publication 60, proposed a value of 2 for low-LET radiation (ICRP 1991). This value was also adopted by UNSCEAR in 1993 (UNSCEAR 1993). While ICRP has confirmed this value in their most recent general recommendations in Publication 103 (ICRP 2007), other expert bodies came to different conclusions. For example, around the same time, the US National Academy of Sciences proposed a value of 1.5 with a range from 1.1 to 2.3 (NRC 2006). While UNSCEAR did not apply a DDREF in their analysis of solid cancers for the UNSCEAR 2006 Report, a linear-quadratic dose–response model was used, which implicitly considers a reduction of risk at low doses (UNSCEAR 2008). DDREF was not directly considered in the report of the French Academy of Sciences, but variations of radiation effects with dose rate were considered as an additional source of uncertainty in the assessment of risks at low doses (Averbeck 2009; Tubiana 2005). Later, the World Health Organisation applied no reduction factor (i.e., a DDREF of 1) in its report on health risk assessment after the Fukushima accident (WHO 2013); and the German Radiation Protection Commission (SSK) opted to abolish the DDREF, corresponding to an implicit value of 1 (SSK 2014). The historical development has been briefly reviewed by Rühm et al. (2015). More recently, UNSCEAR emphasised that while the DDREF is a concept to be used for radiological protection purposes, extrapolation of radiation risks from moderate or high doses and HDR to low doses or LDR may depend on various factors and, consequently, cannot—from a scientific point of view—be described by a single factor (UNSCEAR 2017). For use in probability of causation calculations, values between 1.1 and 1.3 have recently been proposed (Kocher et al. 2018), although the methodology has been questioned (Wakeford et al. 2019).

To review the use of the DDREF for radiological protection purposes, ICRP has initiated Task Group 91 on *Radiation Risk Inference at Low-dose and Low-dose Rate Exposure for Radiological Protection Purposes*. Since 2014, this group is reviewing the current scientific evidence on low dose and LDR effects, including radiation-induced effects from molecular and cellular studies, studies on experimental animals, and epidemiological studies on humans. Results of this activity have been published regularly in the peer-reviewed literature (Haley et al. 2015; Rühm et al. 2015, 2016, 2017, 2018; Shore et al. 2017; Tran and Little 2017; Wakeford et al. 2019; Little et al. 2020).

## Experimental evidence of a dose rate effect

### Experimental setup

The difficulty to study biological effects of different dose rates is well illustrated in Elbakrawy et al. (2019), where micronucleus formation was used as an endpoint. HDR exposure is usually short (less than an hour), whereas LDR exposures can last hours to reach the same dose. For this reason, additional groups were added with HDR irradiation performed in parallel at the start of LDR exposure or at the end. When comparing LDR to HDR effects, they found a difference between LDR and HDR when HDR is done at the beginning of LDR exposure and no difference when performed at the end.

As the time between point/period of exposure and biological endpoint measured impacts the result, performing robust experimentations to understand dose rate effects is challenging. Experimental setups should consider cumulative doses, duration of exposure but also the delay between the start and the end of exposure. For such reasons, experimental design should be well conducted with an appropriate statistical analysis and parallel controls always included.

Due to these difficulties in studying LDR, alternative approaches to detect some differences might be necessary. This includes increasing the dose, making the comparison of LDR and HDR effects less relevant for considering radiological protection. Another possible approach is to apply an adaptive response scheme, where the modulation of the response to a challenging dose due to a priming LDR treatment is used to evidence LDR effects (Satta et al. 2002; Carbone et al. 2009; Elmore et al. 2008).

#### Dedicated infrastructures

In Europe, there are several facilities for in vitro and in vivo exposures to low-dose rates. In the framework of the CONCERT EJP-WG Infrastructure activities, information on some of them has been published in AIR^2^ bulletins (https://www.concert-h2020.eu/en/Concert_info/Access_Infrastructures/Bulletins).

Among European LDR exposure infrastructures, it is worth mentioning the FIGARO facility, located at the Norwegian University of Life Sciences (NMBU), that allows gamma irradiation of up to 150 mice at 2 mGy/h and larger numbers at lower dose rates (AIR^2^ No. 1, 2015). In addition, three facilities with similar features located at the UK Health Security Agency (UKHSA, Harwell), Istituto Superiore di Sanità (ISS, Rome, Italy), and Stockholm University (Sweden) are available for irradiation of cells and/or small animals in a dose rate range 2 µGy/h–100 mGy/h (AIR^2^ No. 11, 2016; AIR^2^ No. 16, 2016). Another platform, the MICADO’LAB, is located at the French Institute for Radiological Protection and Nuclear Safety (IRSN, France). It has been designed to study the effects on ecosystems of chronic exposure to ionising radiation and is able to accommodate experimental equipment for the exposure of different biological models (cell cultures, plants, and animals at dose rates ranging from 5 µGy/h to 100 mGy/h (AIR^2^, No. 19, 2017).

Other interesting facilities where studies at extremely low-dose rates have been carried out are Deep Underground Laboratories (DULs) where dose rates are significantly lower than on the Earth’s surface. Although the main research activity in these infrastructures concerns the search for rare events in astroparticle physics and neutrino physics, DULs offer a unique opportunity to run experiments in astrobiology and biology in extreme environments (Ianni 2021) highlighting biological mechanisms impacted by differences in dose rates. The large majority of data have been collected so far in Italy at the Gran Sasso National laboratory (LNGS, AIR^2^, No. 3, 2015), and in the US at the Waste Isolation Pilot Plant (WIPP). Recently, the interest in this field has been shared by many other DULs where underground biology experiments already started or are planned (SNOLAB Canada, CANFRANC Spain, MODANE France, CJML/JINPING China, BNO Russia, ANDES Argentina). Compared to that at the Earth’s surface, inside DULs, the dose/dose rate contribution due to photons and directly ionising low-LET (mostly muons) cosmic rays can be considered negligible, being reduced by a factor between 10^4^ and 10^7^ depending upon shielding. Radiation exposure due to neutrons is also extremely low, being reduced by a factor between 10^2^ and 10^4^. One further contribution to the overall dose/dose rate can come from radon decay products, but it depends upon the radon concentration, which can be kept at the same levels of the reference radiation environment by a suitable ventilation system. Terrestrial gamma rays represent the major contribution to the dose/dose rate inside the DULs (Morciano et al. 2018b).

#### Dedicated cellular and animal models

MELODI embarked on a large effort beginning around 2008 to collect all archives and tissues from animal irradiation studies done in Europe. The result of this was the European Radiobiological Archive (ERA) that is available to all investigators worldwide, and some of the animal studies included in this collection and database include low-dose rate studies (Birschwilks et al. 2012); www.bfs.de/EN/bfs/science-research/projects/era/era_node.html. In the US, the Department of Energy (DOE) collected archived tissue samples and databases from long-term studies involving approximately 49,000 mice, 28,000 dogs, and 30,000 rats. Data from many of these studies are available on the website janus.northwestern.edu/wololab. While many of these experimental animal studies had been done at low-dose rates and studies were published, the ability to re-analyse them with new statistical and computational approaches allowed for the assessment of the data from new perspectives.

Rodents are particularly radioresistant and wild-type strains will not develop some pathologies of interest, such as atherosclerosis. Therefore, to study some specific mechanisms, the use of transgenic mice can be beneficial for understanding effects observed in humans. Most transgenic mouse studies are limited by the fact that they are imperfect models of the human situation. For example, animals with oncogenic mutations develop caners, but they are often similar but not identical to the human disease (Cheon and Orsulic 2011). Another limitation is that most human disorders that are modelled in transgenic situations have multi-genic causes, but the creation of a transgenic mouse often assumes that a single gene is responsible for the disease. In fact, the transgenic model is a means of testing the molecular consequences of a particular genetic alteration, but the mimicking of disease may be limited. Limitations of models have been pointed out for virtually all animal models that have been studied (Shanks et al. 2009). Finally, one can argue that mice (or indeed any experimental animal) may not adequately model human diseases.

Similar limitations are present in cellular models. Any in vitro experiment is limited by observable endpoints and sensitivity of assays. Despite these limitations, valuable insights can be generated from such experiments, if these findings are not extrapolated beyond the context of the model and experimental setup.

### Dose rate effects at molecular and cellular level

The studies listed in Tables 1, 2, 3, 4 have been selected from the literature to draw some conclusions about radiation biology studies and are explained in some detail below.

**Table 1:** List of relevant publications related to the effect of dose rate on gene expression, protein modification, and cell cycle effects

Type of animal, strain/age, or in vitro cell type	Irradiation details	Outcomes recorded, time post-exposure	Definitive findings	Reference
ML-1 human myeloid leukaemia cell line	γ-Irradiation Doses: 50, 100, 250, 500 mGy Dose rates: 1.68 × 10^2^, 1.44 .× 10^3^ 1.6,8 × 10^4^ and 1.68 × 10^5^ mGy/h	Cell cycle, apoptosis, gene expression (2 h after exposure)	Decrease in the slope of the dose response curve with decreasing dose rate for some genes and no effect on some others	Amundson et al. (2003)
Shewanella oneidensis	Underground vs above ground environment (WIPP, US) Inside DUL: ~ 10^–6^ mGy/h	RNASeq-based transcriptome analysis carried out on early and late-exponential S. oneidensis cultures *(after 5–8-13 17 and 24 h underground)*	Stress response when deprived of background levels of ionising radiation. Down-regulation of ribosomal proteins and tRNA genes; up-regulation of membrane transporters among others	Castillo et al. (2015)
AG1522 normal human skin fibroblast (synchronised in G0/G1; grown in 3D on carbon scaffold)	γ-Irradiation Dose: 100 mGy Dose rates: 3 mGy/h and 30 mGy/h Dose: 4000 mGy Dose rate: 1.2 × 10^3^ mGy/h	miRNA expression pattern *(evaluated at 3 h or 8 h after exposure)*	Evidence of dose, dose rate, and time dependent variations in miRNA expression pattern	Chaudhry et al. (2012)
Chinese hamster V79 cells	Underground vs above ground environment (CJPL, China) Inside DUL: ~ 10^–5^ mGy/h	Whole transcriptome analysis *(after two days of culture underground)*	No significant difference in miRNA. Altered RNA profiles in lncRNAs, mRNAs and circRNAs. Differentially expressed RNAs involved in many pathways including ECM-RI, PI3K-Akt signalling, RNA transport and the cell cycle Cell growth inside DUL could induce transcriptional repression, thus reducing metabolic process and reprogramming the overall gene expression profile in V79 cells	Duan et al. (2021)
A11 mouse hybridoma cells	Underground vs above ground environment (LNGS, Italy) Inside DUL: ~ 10^–6^—~ 10^–5^ mGy/h *(inside a shielded or an unshielded incubator)*	Cell proliferation, caspase-3 activation and PARP1 cleavage (western blot) *(4 weeks of continuous culture in different environments plus 2 weeks above ground)*	No differences in cell proliferation; switch of the over-growth-induced cell apoptosis to autophagy underground, Caspase-3 activation and PARP1 cleavage in cells entering in over-growth status above ground only; p53 activation underground; scarce influence of further gamma ray shielding underground	Fischietti et al. (2020)
Chinese hamster V79 cells	Underground vs above ground environment (LNGS, Italy) Inside DUL: ~ 10^–6^ mGy/h	Gene expression (RT-qPCR) and activities of antioxidant enzymes (GPX, SOD, CAT) *(10 months of continuous culture in different environments plus 6 months above ground)*	Higher degree of defence against endogenous damage in reference conditions: GPX activity significantly decreased underground and remaining at the same level even after further 6 months above ground	Fratini et al. (2015)
Zebrafish AB strain	γ-irradiation Doses: 5.2, 31 Gy Dose rates: 8.7 and 53 mGy/h	Mating, gene expression, health parameters. *(month or years later),* health of F1	Few genes in F1 from different doses overlapping, more overlapping between higher dose 1-month F1 and lower dose 1-year F1	Hurem et al. (2018)
Mouse C57Bl/6	γ-irradiation Doses: 3 × 10^2^, 6 × 10^3^ mGy Dose rates: 0.042 and 0.83 mGy/h	Gene expression analysis *(after 300 days of exposure)*	Differentially expressed gene lists according to dose rate	Kempf et al. (2016)
Chinese hamster V79 cells FD-LSC-1 Laryngeal Squamous Carcinoma Cells	Underground vs above ground environment (CJPL, China) Inside DUL: ~ 10^–5^ mGy/h	Proteome profile, cell proliferation (up to 1 week), morphology (TEM) *(one passage and 2 days or 4 days of growth for V79 or FD-LSC-1 cells respectively)*	Reduced growth rate and changes in the protein profile: up-regulation of ribosomal proteins, spliceosome, RNA transport, and energy metabolism among others	Liu et al. (2020a), Liu et al. (2020b)
Chinese hamster CH0-K1 cells	X-irradiation Doses: up to 15 Gy Dose rates: 1.86 × 10^2^, 1 × 10^3^ mGy/h, 3 × 10^3^ and 6 × 10^3^ mGy/h *(fractionated and continuous exposure)*	Cell survival and cell cycle	Increase survival with decreasing dose rate; accumulation of the cells in G2 during exposure with LDR; delay of DNA synthesis and accumulation of the cells in S/G2 during the exposure with intermediate dose rates (e.g., 3 × 10^3^ mGy/h), Blocks of cell cycle progressing in whole checkpoints (G1/S and G2/M checkpoints) and delay of DNA synthesis during the exposure with higher dose rate (e.g., 6 × 10^3^ Gy/h)	Matsuya et al. (2017) Matsuya et al. (2018)
Paramecium tetraurelia	Underground vs above ground environment (CNRS Moulis, Pyrenees Mountains, France) Inside DUL: in the absence 1,9 × 10^–4^ mGy/h or in the presence of Pb shielding (1,1 × 10^–5^ mGy/h) or after chronic irradiation with a ^60^Co-source (4,6 × 10^–4^ mGy/h). In orbit on Salyut 6 4.6 × 10^–3^ mGy/h) or in balloon flight (in the presence of microgravity) 4 × 10^–3^ mGy/h	Protozoan growth rate *(10 days of culture underground, 2 days in balloon and 4 days on Salyut 6)*	Background radiation or chronic γ-irradiation at very low dose rate can stimulate cell proliferation Stimulation was also observed at high altitude and in space	Planel et al. (1987)
Primary human Lung fibroblasts Bronchial epithelial cells	Underground vs above ground environment (WIPP, USA) Inside DUL: ~ 10^–6^ mGy/h	Western Blot *(after 10 passages underground)*	Upregulation of HSP 90B and HSP 70); expression further upregulated after acute exposure to 10 cGy X-rays	Smith et al. (2011)
Caenorhabditis elegans	Underground vs above ground environment (WIPP, USA) Inside DUL: ~ 10^–5^ mGy/h	Gene expression, rate of larvae growth; egg layering *(after 1 week or 8 months underground)*	More than 100 genes differentially regulated, compared to normal background radiation levels, faster rates of larval growth, and early egg laying	Van Voorhies et al. (2020)
Bacillus subtilis and Escherichia coli	Underground environment (Boulby, UK) Inside DUL: ~ 10^–7^ -10^–5^ mGy/h *(in the absence or in the presence of a gamma source)*	Bacterial growth and susceptibility to stress (UVC exposure)	No significant effect on bacterial growth from exposure to radiation doses ranging from 0.01 times the levels of background radiation to 100 times that background No preconditioned susceptibility to stress was observed in the bacterial strains grown in sustained low radiation	Wadsworth et al. (2020)
Drosophila melanogaster	Underground vs above ground environment (DULB-4900, BNO, Russia) Inside DUL: ~ 10^–5^ mGy/h	Transcriptome analysis after one development cycle *(14 days)*	Gene expression changes in several genes (77) in response to underground environment	Zarubin et al. (2021)
Human umbilical vein endothelial cells (HUVECs)	γ-irradiation Dose: ~ 2 × 10^3^ mGy and ~ 4 × 10^3^ mGy, depending on the dose rate Dose rates: 1.4, 2.4 and 4.1 mGy/h Chronic exposure for up to 10 weeks	Transcriptome and proteome analysis, *cell proliferation and senescence*	Dose-rate-dependent signatures of proteomic changes involved mainly in the PI3K/Akt/mTOR pathway and oxidative stress; gene expression profiling demonstrating an early stress response characterised by the expression of inflammation-related genes possibly activating radiation-induced premature senescence via the IGFBP5 signalling pathway	(Rombouts et al. 2014; Yentrapalli et al. 2013a, 2013b)

**Table 2:** Relevant references related to the effect of dose rate on mutation induction

Type of animal/strain or in vitro cell type	Irradiation details	Outcomes recorded, time post-exposure	Definitive findings	Reference
TK6 and WTK1 human lymphoblasts	γ-irradiation Doses: up to 6 Gy Dose rates: 27, 67, 143 mGy/h and 6 × 10^4^ mGy/h	HPRT mutations, survival, cell growth, cell cycle distribution,	Compared with acute doses, the low dose rates protected against mutation induction at the *hrpt* locus in WTK1, but protection was inversely related to dose rate. Slight inverse dose-rate effect in TK6, with mutation induction at the lowest dose-rate exceeding that at acute exposures	Amundson and Chen (1996)
human lymphoblastoid WIL2-NS cells	γ-irradiation Doses: up to 5 Gy Dose rates: 170 mGy/h and 3 × 10^4^ mGy/h	HPRT mutations and molecular changes in the HPRT gene	Dose rate effect on cell death; HPRT mutant frequency lower after LDR compared to HDR LDR is as efficient as HDR to produce HPRT deletions	Furuno-Fukushi et al. (1996)
Chinese hamster V79 cells	Underground vs above ground environment (LNGS, Italy) Inside DUL: 10^–6^ mGy/h	Spontaneous and radiation-induced mutation frequency at the HPRT locus *(10 months of continuous culture in different environments)*	Increased mutation frequency at the *hprt* locus before (spontaneous level) and after irradiation with X-ray doses in cultures kept for 10 months underground respect to those above ground Further significant increase of spontaneous HPRT mutant frequency in cells kept for 10 months underground after another 6 months above ground	Fratini et al. (2015)
Drosophila melanogaster (wt Canton S and a mutant strain defective in the excision repair function, (y mei-9a v f y)	X-irradiation Doses: 0.2 and 10 Gy Dose rates: 3 × 10^3^ and 3 × 10^4^ mGy/h	Sex-linked recessive lethal assay using immature sperm	Mutation frequency in the sperm irradiated with a low dose at a low-dose rate significantly lower than that in the sham-irradiated group	Koana et al. (2007)
Human telomere reverse transcriptase (TERT)-immortalised fibroblast	X-irradiation Doses: up to 5 Gy Dose rates: 18 mGy/h and 12 × 10^4^ mGy/h	Mutation induction, PCR analysis of HPRT mutants; survival and micronucleus induction	Less HPRT mutation and less deletions after LDR compared to HDR; smaller size of the deletions after LDR	Nakamura et al. (2005)
Transgenic *gpt* delta mice	γ-Irradiation Doses and dose rates: 0.75 mGy/h (for 483 consecutive days, i.e.,up to ~ 8.7 Gy, ~ 60 mGy/h (for 2, 4 and 8 days, i.e., up to ~ 11.5 Gy), and ~ 5.5 × 10^4^ mGy/h (for 2, 4 and 8 days, i.e., up to ~ 1 × 10^4^ Gy	Transgenic assay in spleen and liver (mice contain bacterial genes in their genome, which can be assayed for mutations by using bacterial systems)	Mutation induction rate-dependent on the dose rate; it is higher in the spleen than in the liver at the medium-dose rate but similar in the two tissues at the high and low-dose rates. Deletion without any sequence homology at the break point elevated in spleen after HDR irradiation (tissue-specific response)	Okudaira et al. (2010)
S. cerevisiae	Underground vs above ground environment (LNGS, Italy) Inside DUL: 10^6^ mGy/h	Susceptibility to treatments with high doses of a radiomimetic chemical agent (MMS) in terms of mitotic intergenic recombination *(after 120 generation, i.e., 1 week)*	Higher frequency of recombination in yeast cells grown underground respect to those grown above ground	Satta et al. (1995)
TK6 human lymphoblasts	γ-Irradiation Doses: 0.5, or 1.0 Gy Dose rates: 1.4, 5.0, 15.0, and 30.0 mGy/h	Cell growth, frequency of thymidine kinase (TK) mutants, and of chromosomal aberrations in painted chromosomes 2, 8, and 14	Clear lack of dose rate effect on the frequency of mutants and stable-type chromosomal aberrations (mainly translocations), with a dose rate effect on cell growth and unstable-type aberrations (dicentrics and breaks)	Shakeri Manesh et al. (2014)

**Table 3:** Relevant references related to DNA and chromosome damages

Type of animal, strain/age, or in vitro cell type	Irradiation details	Outcomes recorded, time post-exposure	Definitive findings	Reference
Lens epithelial cells from irradiated C57BL/6 mouse	γ-Irradiation Doses: 0.5, 1 and 2 Gy Dose rates: 0.23 (only for 0.5 Gy), 1.05 and 5 mGy/h	53BP1 foci *(measured at 4 and 24 h)*	The number of 53BP1 foci persisting in the mouse lens samples after γ-radiation exposure increased with decreasing dose-rate at 4 and 24 h	Barnard et al. (2019)
Human lymphocytes exposed in vitro	γ-Irradiation Doses: from 0.5 to 4 Gy Dose rates: 1.25 × 10^2^, 5 × 10^2^, 1 × 10^3^ and 1.78 × 10^5^ mGy/h	Chromosomal abnormalities, MN induction	A trend towards linearity when the dose rate increases. No curvature for the lower dose rate	Bhat and Rao (2003a)
Mouse C3H/HeN	γ-Irradiation Doses: up to 8 Gy or 80 Gy Dose rates: ~ 0.83 and ~ 8.3 mGy/h	Chromosomal abnormalities	Linear increase in number of dicentrics in spleen cells over time for both dose rates. Unstable aberrations per cell show a spike at 1 Gy cumulative dose for 20 mGy/d group, data points not significant however	Braga-Tanaka et al. (2018a) Nakajima et al. (2008)
TK6 human lymphoblasts	Underground vs above ground environment (LNGS, Italy) Inside DUL: 10^–6^ mGy/h	MN induction after acute irradiation with a challenging dose of 2 Gy X-ray *(after 6 months of continuous culture)*	Increase of radiation-induced MN frequency in two sister cultures kept in reduced environmental radiation background	Carbone et al. (2009)
Mouse C57BL6/6Crl	γ-Irradiation Doses: 100, 200, 500 or 1000 mGy Dose rates: 1.4 mGy/h and ~ 2.2 × 10^4^ mGy/h	MN induction in bone marrow cells evaluated after 3 h and 3 weeks after exposure	Slight differences between MN levels induced by LDR and HDR, arguing against a DDREF factor of 2 for MN induction as marker for genotoxicity	D'Auria Vieira de Godoy et al. (2021)
HF19 human fibroblasts	X-Irradiation Doses 0,1 Gy and 1 Gy Dose rates: 1.86 and 2.5 × 10^4^ mGy/h	MN induction for radiation-induced genomic instability, oxidative stress *Time post-exposure: 1.5 h for ROS and immediately post-exposure (40 h post-cytochalasin B treatment) and after 15 (10 population doublings) and 20 days (20 population doubling)*	Immediately after exposure no clear-cut dose rate effect whatever the dose considered After 15 and 20 days still a higher yield of MN in all exposed groups compared to control; no difference across all exposed groups but a slightly enhanced yield after HDR vs LDR	Elbakrawy et al. (2019)
Zebrafish Adults exposed for 10 days; progeny exposed to 4–5 days	γ-Irradiation Doses: from 120 mGy to 12 Gy Dose rates: 0.5, 5 and 50 mGy/h for 10 days	DNA damage (comet assay), health status (progeny survival; ROS production)	DNA damage and apoptosis at 570 mGy/d	Gagnaire et al. (2015) Guirandy et al. (2019)
Mouse C57BL/6, 12-week-old	γ-Irradiation Doses: 60, 300, 1,500, 7,500 mGy Dose rates: 8.3 × 10^–2^, 0.42, ~ 2.1 × 10^3^, ~ 10.4 × 10^3^ for 30 days^* c*^	Muscle effects, immediately after ionising radiation or 3 months after	Satellite cells isolated from muscles had lower numbers when isolated from animals after 3 months at all dose rates	Masuda et al. (2015)
Mouse SWR x C57BL/ (M + F)	γ-Irradiation Doses and dose rates: 0.21 mGy/h (150- 450 mGy) 0.83 mGy/h (600–1,800 mGy) 1.66 mGy/h (1,200- 3,600 mGy)	Micronucleated polychromatic erythrocytes (MPCE) and micronucleated normochromatic erythrocytes (MNCE) Translocations by FISH Duration of exposure 30–60-90 days Blood sampling 2 days post-end of exposure	Dose rate effect between acute and chronic exposure but no significant difference among translocation frequencies between the three chronic dose rates	Sorensen et al. (2000)
Mouse C3H/HeN	γ-Irradiation Doses and dose rates: ~ 4.2 × 10^–2^ mGy/h (up to 0,7 Gy), 0.83 mGy/h (up to 8 Gy) ~ 16.6 my/h (up to 8 Gy), and 5.34 × 10^4^ mGy/h (0.25- 3 Gy)	Dicentric (Dic) and translocations (Tr) measured in splenic lymphocytes at the end of continuous exposure	Reduced yield of Dic and Tr with reduced dose rate Positive dose rate effect when comparing 20 mGy/day and 1 mGy/day but driven by the high doses Higher Translocations yield after 1 mGy/d exposure compared to the control group	Tanaka et al. (2013) Tanaka et al. (2014)
Mouse C57BL/6	X-irradiation Doses 1.1; 2.2 and 4.4 Gy Dose rates: 1.96 × 10^2^ and 6.2 × 10^4^ mGy/h	Mouse blood ymphocyte response in terms of DSB (γ-H2AX 24 h post-start of exposure), apoptosis (Tunel assay at 24 h), MN induction after 24 h and 7 days	Higher yield of foci and apoptosis after HDR exposure for 1.1 and 2.2 Gy DSB less repaired after HDR No significant dose-rate effect for MN induction across the dose range examined: link to a possible lack of dose rate effect on repair process	Turner et al. (2015)
Human MSC	γ-irradiation Doses: up to 300 mGy Dose rates: 6 mGy/h and 1.8 × 10^3^ mGy/h	γ-H2AX and pATM foci *(measured for up to 6 h)*	Non-linear response for both types of foci after chronical exposure with a threshold which is not observed after acute exposure	Ulyanenko et al. (2019)

**Table 4:** Relevant references related to epigenetics and ageing

Type of animal, strain/age, or in vitro cell type	Irradiation details	Outcomes recorded, time post-exposure	Definitive findings	Reference
Chinese hamster V79 cells	Underground vs above ground environment (LNGS, Italy) Inside DUL: ~ 10^–6^ mGy/h	Spontaneous mutation frequency at the hprt locus, gene expression (RT-qPCR) and activities of antioxidant enzymes (GPX, SOD, CAT) *(10 months of continuous culture in different environments plus 6 months above ground)*	Involvement of epigenetic regulation suggested by the persistence of high mutation frequency and low GPX enzymatic activity after the sister cultures, cultured underground for 10 months, are kept for further 6 months above ground	Fratini et al. (2015)
MouseC57BL, 45 days-old males and females	X-irradiation Total dose: 500 mGy Dose rate: 7.2 × 10^3^ mGy/h Repeated exposure (chronic) 50 mGy each day for 10 days Acute exposure: 500 mGy given.at day 10 of treatment of the chronic group	Methylation analysis and gene expression in muscle and liver tissues	Global methylation in liver higher than in muscle, both in male and females Pronounced changes in the locus specific methylation patterns documented only in p16^INKa^ of chronically irradiated males, showing a significant de novo methylation. Less pronounced de novo methylation of p16^INKa^ in the liver tissue of exposed females Chronic low-dose radiation exposure acts as a more potent inducer of epigenetic effects than acute exposure	Kovalchuk et al. (2004)
Zebrafish and Atlantic salmon	γ-Irradiation Dose rates: Adult zebrafish irradiated during gametogenesis for 27 days at 8.7 and 53 mGy/h (total doses: ~ 5.6 and ~ 34 Gy Atlantic salmon embryos continuously exposed from one-cell to early gastrula stage (30 days) at 1, 10, 20 and 30 mGy/h	Ovaries of exposed adult Zebrafish showed H3 enrichment tended to be correlated with H3K4me3 enrichment, the lowest enrichment occurring at the highest dose rate Similar enrichment was observed in F1, no or very small differences were instead observed in F2 generation For Atlantic salmon, a pronounced enrichment of H3K4me3 was induced by 30 mGy/h gamma radiation; no significant changes were seen at the lower dose rates	Ionising radiation can affect chromatin structure and organisation in a dose rate-dependent manner; these changes can be detected also in F1 offspring, but not in subsequent generations	Lindeman et al. (2019)
Primary human keratinocytes and fibroblasts	γ-irradiation Doses: up to ~ 3.4 Gy (chronic exposure for 7 days) Dose rate range: 6–20 mGy/h X-irradiation Dose: 4 Gy (acute exposure) Dose rate: 3 × 10^4^ mGy/h	Histone levels, gene expression, ATM phosphorylation	Reduction of histone levels in chronically irradiated cells occurring mainly though reduced transcription, not protein degradation	Lowe et al. (2020)
VH10 normal human fibroblasts	γ-irradiation Doses: 14.4 Gy and 43.2 Gy (chronic exposure for 120 days) Dose rates: 5 and 15 mGy/h	Proliferation and senesce, proteomic profile	Chronic exposure above 5 mGy/h in dividing fibroblasts causes premature senescence (proteomic profile similar to replicative senescent fibroblasts)	Loseva et al. (2014)
Human umbilical vein endothelial cells (HUVECs)	γ-irradiation Doses: from ~ 2 × 10^3^ mGy to ~ 7 × 10^3^ mGy, (depending on the end point) Dose rates: 1.4, 2.1 and 4.1 mGy/h Chronic exposure for up to 16 weeks	Cell proliferation, senescence, vascular network formation, transcriptomic and proteomic profiles Combined experimental and modelling approaches	Different dose rates accelerate the onset of the senescent status, shorten the life span, modify the sustaining mechanisms leading to alterations in the proliferative status or the vascular network formation Strong dose-dependence in the measured endpoints, in some cases with a clear dose threshold (as for the loss of vascular network formation capability)	(Babini et al. 2022; Rombouts et al. 2014; Yentrapalli et al. 2013a, 2013b)

#### Gene expression, protein modification, and cell cycle effects

There have been several studies that have examined gene and protein expression in animals prone to particular conditions (either genetically engineered or having background genetic mutations) using LDR exposures (Ina and Sakai 2005; Ebrahimian et al. 2018a; Mathias et al. 2015; Ishida et al. 2010). These all showed differences in gene expression patterns between LDR and HDR exposed mice and differences in lymphocyte activation and cytokine expression. A tissue-specific response has been identified among tissues linked to the difference in DNA damage repair processes (Taki et al. 2009). Changes in cell cycle progression have also been reported to show dose rate effects with increases in survival, accumulation of cells in G2 phase following LDR, and delays of DNA synthesis (Matsuya et al. 2018, 2017).

In addition to mice, other animals and cultured cells have been analysed for gene expression and protein modifications after LDR exposures (see Table 1).

Alterations in several genes related to ribosomal proteins, membrane transport, respiration, and antioxidant regulation for increased reactive oxygen species (ROS) removal were also observed in experiments carried out inside DUL on mammalian cell cultures and organisms (Smith et al. 1994; Fratini et al. 2015; Van Voorhies et al. 2020; Liu et al. 2020b; Zarubin et al. 2021; Castillo et al. 2015). In a recent paper, Fischietti et al. (2020) reported that *pKZ1 A11 mouse hybridoma* cells growing underground at the LNGS display a qualitatively different response to stress; induced by over-growth with respect to the external reference laboratory. Analysis of proteins known to be implicated in the cell stress response has shown that after 96 h of growth, the cell culture kept in the external laboratory shows an increase in PARP1 cleavage, an early marker of apoptosis, while the cells grown underground present a switch from apoptosis toward autophagy, which appears to be mediated by p53. This behaviour is not affected by a further reduction of the gamma radiation dose by shielding. Interestingly, this effect reverted when, after 4 weeks of underground culture, cells were moved to the reference radiation environment for 2 more weeks, indicating a plasticity of cells in their response to the low-radiation environment. Transcriptomic and methylation analysis are presently underway to understand the genetic and epigenetic bases of the observed effects. Of crucial importance is also trying to identify the component(s) of the radiation spectrum triggering the biological response.

Overall, the data suggest that biological systems are very good sensors of changes in environmental radiation exposure, in particular regarding dose rate effects, and also support the hypothesis that environmental radiation contributes to the development and maintenance of defence response in cells and cultured organisms. Nevertheless, it should be noted that extrapolation from experimental cell or animal models to humans is very challenging, because it depends upon many parameters, including the model, endpoints, and radiation exposure type. More work is needed to determine which models are best for certain human endpoints.

#### Mutation

Early studies were done by William and Leanne Russell at Oak Ridge National Laboratory in the 1960s examining the development of coat colour mutations in mice following exposure to gamma rays (Russell 1963, 1965; Russell et al. 1958). This work is now considered classic and helped establish that LDR exposure (8 mGy/min or less) induced fewer hereditary mutations in mice compared to the same dose administered at HDR. Later, this work was confirmed by Lyon et al. (1979) and Favor et al. (1987).

In contrast, an inverse dose rate effect for survival was originally observed initially in both S3HeLa and V79 cells in culture (Mitchell et al. 1979). This initial work was expanded to include experiments on mutation induction by LDR carried out in the 1990s. Among them, the work of Amundson and Chen (1996) reported an inverse dose rate effect in syngeneic human TK6 and p53-deficient WTK1 lymphoblastoid cell lines exposed to continuous LDR γ-irradiation. These data have been interpreted on the basis of the assumption that at low-dose rates, cell cycling can cause mutated cells to progress to resistant phases before they are killed, resulting in previously resistant surviving cells progressing to a sensitive part of the cycle, where they can undergo mutagenesis (Brenner et al. 1996). Different results have been obtained by Furuno-Fukushi et al. (1996), who using WIL2-NS human lymphoblasts did not find an inverse dose rate effect.

The studies cited above, along with other published data on HPRT mutation in various rodent and mammalian cells, were re-analysed by Vilenchik and Knudson (2000). They showed that for both somatic and germ-line mutations, there is an opposite, inverse dose rate effect, with reduction from low to very low-dose rate, the overall dependence of induced mutations being parabolically related to dose rate, with a minimum in the range of 0.1 to 1.0 cGy/min (60 to 600 mGy/h). They suggested that this general pattern could be attributed to an optimal induction of error-free DNA repair in a dose rate region of minimal mutability. This study also predicts on a quantitative level that induction of DNA repair and/or antioxidant enzymes by radiation depends not only on the level, but also on the rate of production, of certain DNA lesions and ROS, with an optimal response to an increase of 10–100% above the “spontaneous’’ background rates.

In human telomere reverse transcriptase (TERT)-immortalised fibroblast cells obtained from normal individuals, Nakamura et al. (2005) demonstrated that the genetic effects (HPRT mutation induction and size of the deletions induced) of low-dose rate radiation were much lower in nonproliferating human cells than those seen after high-dose rate irradiation, suggesting that LDR radiation-induced damage was repaired efficiently and correctly with a system that was relatively error-free compared to that repairing damage caused by HDR irradiation.

Koana et al. (2007), investigated mutation induction in Drosophila spermatocytes after low and high X-ray doses delivered at two different dose rates (0.05 Gy min and 0.5 Gy/min). They obtained evidence of error-free DNA repair functions activated by low dose of low-dose-rate radiation (0.2 Gy; 0.05 Gy/min) able to repair spontaneous DNA damage (detectable in the sham sample). This was not observed at the higher dose rate. After a high-dose exposure (10 Gy), a significant increase in the mutation frequency with respect to the sham-irradiated group was observed, independently on the dose rate (0.5 Gy/min or 0.05 mGy/min). The authors proposed the presence at low-dose rate of a threshold between 0.2 and 10 Gy below which no increase in mutation frequency is detected.

Mutation experiments have also been carried out at the LNGS underground laboratory. The first evidence was obtained in yeasts, which showed a high frequency of recombination when grown underground as compared to above ground (Satta et al. 1995). Afterwards, using Chinese hamster V79 lung cells, an increased mutation frequency at the *hprt* locus was observed before (spontaneous level) and after irradiation with challenging X-ray doses in cultures kept for 10 months underground compared to those kept above ground (Satta et al. 2002), suggesting more damage at a very low-dose-rate exposure. Further long-term experiments provided evidence against mutant selection and in favour of the involvement of epigenetic regulation in the observed increase of spontaneous *hprt* mutation frequency after 10 months of growth underground and other 6 months above ground (Fratini et al. 2015). Biochemical measurements of antioxidant enzymatic activity have shown that cells maintained in the presence of “reference” background radiation are more efficient in removing ROS than those cultured in the underground environment.

A summary of the experiments described here can be found in Table 2.

#### DNA and chromosomal damages

The dose rate effect on chromosomal aberrations (CA) after ex vivo blood exposure is well known, since Scott et al. (1970) reported fewer chromosomal aberration yield when the dose rate decreases. More recent data (Bhat and Rao 2003b) have confirmed the linear-quadratic response for chromosomal damage induction (micronuclei) after acute (high does rate) exposure (178.2 Gy/h) and the trend to a linearity when the dose rate decreases to reach a linear dose response for the lower dose rate (125 mGy/h).

However, the in vitro studies used to establish this dose rate effect have mainly been performed using a dose rate of the order of Gy/min, which is much higher than that received in the environment or by workers, and is more in the area of high- and medium-dose rate as defined by UNSCEAR.

In vitro experiments have shown an increase in radiation-induced micronuclei frequency (2 Gy challenging dose) in TK6 lymphoblasts after six months of continuous growth in reduced environmental radiation background at the LNGS underground laboratory as compared to the external reference laboratory at the ISS (Carbone et al. 2009).

In vivo experimental studies have measured dicentrics and translocations produced in mice after much lower dose rates starting from 1 mGy/day. One of them compares induction of chromosomal damage after exposure to ~ 1 mGy/day, ~ 20 mGy/day, ~ 400 mGy/day (16.7 mGy/h) with 890 mGy/min (53,400 mGy/h) as an acute group; cumulative doses ranged from 125 mGy to 8 Gy. The dose rate effect on both types of CAs was confirmed and a dose rate effect was even measured when comparing translocations and dicentrics induced after 20 mGy/day and 1 mGy/day exposure but also with a higher translocations yield after 1 mGy/d exposure compared to the control group (Tanaka et al. 2013, 2014). This was also confirmed in another study (Sorensen et al. 2000) comparing 50 mGy/day with 200 mGy/day and 400 mGy/day (duration of exposure up to 90 days with cumulative doses up to 3.6 Gy). No difference among the chronically exposed group was identified but again a difference from the acute exposed group was detected.

The main limitation of both studies is that cumulative doses and/or duration of exposures are different among the groups. When the analysis was restricted to doses more compatible with what could be received in the whole exposure time of an individual (between 0.3 and 1 Gy), then the difference in dose rate was not so important and, consequently, it is very difficult to draw any conclusions on whether there is or not a dose rate effect.

Some DDREFs have been derived from the above studies based on the modelling of dose rate relationship without excluding the higher doses which drives the beta coefficient of the curves. Based on Tanaka et al.’s (2013) data sets, the DDEF values calculated ranged from 2.3 (translocation for 100 mGy) to 17.8 (dicentrics for 1000 mGy).

Other in vivo studies do not find a dose rate effect. No significant dose rate effect for micronuclei induction frequency across the dose range has been observed as examined by Turner et al. (2015) in spite of approximately 300 times difference between the two dose rates compared of 1.03 Gy/min and 186 mGy/h, but these dose rates are much higher than those used in Tanaka et al. (2013) and close to the in vitro studies.

A summary of selected experiments can be found in Table 3.

#### Epigenetics and ageing

Epigenetics is the study of the mitotically and/or meiotically heritable changes in gene activity and transcript architecture, including splicing variation, that cannot be explained solely by changes in DNA sequence. Epigenetic alterations include DNA methylation, chromatin remodelling, histones’ modifications, and microRNA-regulated transcriptional silencing. Their impact appears to be greater with low-dose rates than acute exposure. Genetic and epigenetic mechanisms appear to have their common origin in the radiation-induced ROS and/or reactive nitrogen species. Both mechanisms contribute to the complex response to radiation exposure and underlie non-linear phenomena (e.g., adaptive responses), particularly relevant at low doses/LDR (Vaiserman 2011; Schofield and Kondratowicz 2018; Belli and Tabocchini 2020).

Kovalchuk et al. reported different patterns of radiation-induced global genome DNA methylation in C57/Bl mice after whole-body exposure to 50 mGy/day over a period of 10 days or an acute X-ray irradiation of 500 mGy. This was found in the liver and muscle of exposed male and female mice, with hypomethylation induced in the muscle of both males and females, but not in the liver tissue. Sex- and tissue-specific differences in methylation of the p16INKa promoter were also observed (Kovalchuk et al. 2004). A role of DNA hypermethylation was suggested to be involved in adaptive response induced by long-term exposure to low-dose γ-irradiation of human B lymphoblast cells. A novel mechanism of radiation-induced adaptive response was proposed involving the global genomic DNA methylation which is crucial for cell proliferation, gene expression, and maintenance of genome stability, but also important for maintenance of chromatin structure and regulation of cellular radiation response (Ye et al. 2013).

Other laboratory and field studies have demonstrated changes in overall DNA methylation and trans-generational effects in organisms, including C. elegans and zebrafish, exposed chronically to ionising radiation (Kamstra et al. 2018; Horemans et al. 2019).

Post-translational modifications on histone proteins controlling the organisation of chromatin and hence transcriptional responses that ultimately affect the phenotype have been observed in fish (zebrafish and Atlantic salmon). Results from selected loci suggest that ionising radiation can affect chromatin structure and organisation in a dose rate-dependent manner, and that these changes can be detected in F1 offspring, but not in subsequent generations (Lindeman et al. 2019).

A peculiar aspect of low dose/LDR exposure is that related to the ionising radiation background. Experiments carried out in DULs using cultured cells or organisms suggest that very low levels of chronic exposure, such as the natural background, may trigger a defence response without genetic change, therefore mediated by epigenetic mechanisms (Fratini et al. 2015; Morciano et al. 2018a, 2018b). This explanation is consistent with the hypothesis of the epigenetic origin of responses such as adaptive response and non-targeted effects.

Chronic radiation exposure of primary human cells to gamma-radiation between 6 and 20 mGy/h over 7 days has been demonstrated to reduce histone levels in a dose rate-dependent manner (Lowe et al. 2020). This is linked to the induction of senescence, which is a key cellular outcome of LDR radiation exposure (Loseva et al. 2014). Since senescence is linked to many age-related pathologies, including cardiovascular disease, the increase of senescent cells with a tissue following chronic radiation exposure would be expected to cause premature ageing. However, there is contradictory evidence. First, some animal experiments have shown (albeit rarely) that lifespan has been extended by chronic radiation exposure, albeit at much lower dose rates than these in vitro experiments. Second, the development of an epigenetic clock to measure biological age using changes in DNA methylation (Horvath 2013) has demonstrated that cells cultured while being exposed to dose rates between 1 mGy/h and 50 mGy/h do not show any difference in epigenetic age (Kabacik et al. 2022).

Studies specifically showing dose rate dependence of epigenetic effects are summarised in Table 4.

#### Discussion

Dose rate effects are evident when examining gene expression and protein modifications; nevertheless, a comparison of such studies demonstrates that there are broad differences in gene and protein expression depending upon cell type, radiation conditions, culture conditions, and others. This suggests that the endpoints of gene/protein expression may be sensitive markers of radiation effects, but that they are influenced by many factors making broad application of the results difficult. In addition, most changes are observed shortly after exposure and cannot necessarily be linked to adverse health effects among humans. Similarly, no clear response can be highlighted from epigenetic studies. In vitro and in vivo studies have investigated the dose rate effect on mutations, allowing meta-analyses to be conducted, which broadly support an inverse dose rate response.

The study of LDR with in vitro models is limited as such models can only be exposed for durations from minutes to weeks and late endpoints might be affected by too many parameters. The impact of dose rate generally observed shortly after exposure might not be reflected on later endpoints.

#### Conclusions from dose rate effects at molecular and cellular level

For chromosomal aberrations, a dose rate effect is well described but only clear for cumulative doses over 0.5 Gy when an increase in aberrations is observed. An inverse dose rate effect has been reported consistently for limited endpoints including mutations and cell survival.

Overall, evidence from studies at cellular and molecular level suggests potential positive cellular effects and minimal adverse genetic effects at low-radiation dose rates, as long as a total cumulative dose remains low.

### Dose rate effects on lifespan, cancer, and non-cancer endpoints

Many endpoints are impossible to study in vitro; therefore, it is necessary to use animal models to observe specific end points and systematic effects. Here, we describe radiation dose rate and its effects on lifespan, cancer, and non-cancer endpoints. Again, key studies we have considered are summarised in Table 5.

**Table 5:** Relevant references related to lifespan, cancer endpoints, and non-cancer effects

Type of animal, strain/age	Irradiation details	Outcomes recorded, time post-exposure	Definitive findings	Reference
*Wild-type animals*				
Mouse, A/J	γ-irradiation exposure over 3 weeks Doses: 60 and 600 mGy Dose rates 0.12 and 1.2 mGy/h	Lung cancer development at 46 weeks	60 mGy alone is protective, increased adenoma and carcinoma for combined 60 mGy–benzo[a]pyrene, 600 mGy combined less cancer	Bruce et al. (2012)
Mouse, C57BL/6 J	X-irradiation for 4 or 8 weeks Doses: 350, 700 and 1,400 mGy Dose rates 12.5 and 25 mGy every other day for group 1. Dose rates 12.5 and 25 mGy weekly for group 2	Diabetic nephropathy evaluation	Less kidney fibrosis, protection by 12.5 or 25 mGy for 8 weeks best Lower creatinine and connective tissue growth factor	Cheng et al. (2014) Cheng et al. (2018)
Dog, beagle	γ-irradiation for the entire duration of life Dose rates: LDR: 0.125; 0.31; 0.78; 1.6; 3.12 mGy/h HDR: 11; 15,6; 27 mGy/h	Myeloproliferative diseases, lifespan, tumours	Dose rate-dependent life shortening Hematopoietic failure for all dose rates except at 0.125 mGy/h	Fliedner et al. (2012);
Mouse, C57BL/6JJcl females mated with C3H/HeNJcl males, 6-week-old	γ-irradiation Doses of 360, 3,600, 7,200 mGy Dose rates: 0.8, 8.3 and 17 mGy/h	Pregnant mice exposure form day 0 to day 18 of gestation	Foetus size and foetal organ sizes, decreased in all groups at all doses and dose rates; some dose rate differences were noted with less impact at the lower dose rates	Gulay et al. (2018)
Drosophila melanogaster	Underground vs above ground environment (LNGS, Italy) Inside DUL: ~ 10^–5^ mGy/h	Life span, reproductive capacity, response to genotoxic stress *(up to 3 months underground)*	Maintenance in DUL environment prolongs the life span, limits the reproductive capacity of both male and female flies as well as the response to genotoxic stress Effects observed as early as after one generation time (10–15 days) and retained in a trans-generational manner (at least for 2 more generations)	Morciano et al. (2018a) Morciano et al. (2018b)
Lake whitefish (Coregonus clupeaformis)	Underground vs above ground environment (SNOLAB, Canada) Inside DUL: ~ 10^–6^ mGy/h	Timing of hatch, survival, increase in body length and body weight *(up to about 5 months underground)*	Incubating embryos within SNOLAB can have a subtle yet significant effect on embryonic growth and development. (significant increase in body length and body weight of up to 10% observed in embryos reared underground)	Pirkkanen et al. (2020)
Mouse, B6C3F1	γ-irradiation exposure for 400 days Doses 8,000, 400 and 20 mGy Dose rates: 0.002; 0.05 and 0.8 mGy/h	Lifespan, neoplasia	Body weight increase for 0.05 and 0.08 mGy/h Increase in the number of multiple primary neoplasms per mouse after 0.8 mGy/h (total dose 8 Gy)	Tanaka et al. (2007) Braga-Tanaka et al. (2018b)
Caenorhabditis elegans	Underground vs above ground environment (WIPP, USA) Inside DUL: ~ 10^–5^ mGy/h	Rate of larvae growth; egg layering, gene expression *(1 week underground or 8 months underground)*	Faster rates of larval growth, a faster rate of early egg laying, and more than 100 genes were differentially regulated, compared to normal background radiation levels	Van Voorhies et al. (2020)
Mouse, BCF1	γ-irradiation exposure for 400 days Doses: 20, 400 or 2000 mGy Dose rates: 2 × 10^–3^, 4.6 × 10^–2^, 0.87 mGy/h	Tumour incidence	Similar incidence for similar doses vs. Tanaka; fractionated radiation decreases incidence for any dose	Zander et al. (2020)
Transgenic animals				
Mouse, ApoE^−/−^ male on C57BL/6 J; 8 weeks	γ-irradiation Doses: 69, 161 mGy Dose rates: 1.2 × 10^–2^ and 2.8 × 10^–2^ mGy/h	Pathology of atherosclerosis	Macrophages and gene expression show adaptive response Cytokines (IL-4, -10, -13 and -18), catalase, SODs, and CTAT1 upregulated	Ebrahimian et al. (2018a)
Mouse, MRL-lpr/lpr female, 5 weeks old	γ-irradiation Dose rates: 0.35 and 1.2 mGy/h	Life expectancy	Best improvements in life expectancy with 1.2 mGy/h at 5 weeks, some with 0.35 mGy/h	Ina and Sakai (2005)
Mouse, ApoE^−/−^ on C57BL/6 J; 8-week-old	γ-irradiation Doses: 25–2000 mGy Dose rates: 60 mGy/h and 9 × 10^3^ mGy/h	Heart pathology and aortic atherosclerosis	Adaptive response at doses up to 0.5 Gy; increased capillary density, evident at low- and high-dose rates Decreased inflammatory vascular markers; changes of collagen IV and Thy-1 tissue levels	Mathias et al. (2015)
Mouse, ApoE^−/−^ on C57BL/6 J; irradiated at 2 or 8 months of age	γ-irradiation Doses: 25, 50, 100 and 500 mGy Dose rates: 60 mGy/h and 9 × 10^3^ mGy/h	Pathology of aortic atherosclerosis exposure at 2 months and examined at 3 or 6 months later; exposure at 8 months and examined at 2 or 4 months later	Disease slowed down by 25 or 50 mGy delivered at 60 mGy/h	Mitchel et al. (2011)
Female mouse C57BL/6 J ApoE^−/−^ Trp53^+/−^ mice irradiated at 2 or 7 months of age	γ-irradiation Doses: 25, 50, 100 and 500 mGy Dose rates: 60 mGy/h and 9 × 10^3^ mGy/h	Aortic root lesions Exposure at 2 months and examined at 3 or 6 months later; exposure at 7 months and examined at 2 or 4 months later	When exposed at early stage, decrease of lesion progression after doses as low as 25 mGy either after the high or the low-dose rate, Detrimental effect for both dose rates when exposed at later stage	Mitchel et al. (2013)

#### Lifespan and cancer-related end points

The development of a meta-analysis of animals from large-scale databases permitted a reassessment of the DDREF as had been reported by the BEIR VII Committee in the US (Haley et al. 2015). It determined that the values used were based on the use of low doses without direct comparisons of dose rate, so were considered inaccurate. These studies used lifespan as an endpoint. More recent comparisons used rodents in a large-scale multi-year single study that were exposed to protracted vs acute exposures. Considering cancer mortality, the authors concluded that the ratio of HDR to LDR (< 5 mGy/h) gamma dose–response slopes, for many tumour sites was in the range 1.2–2.3, albeit not statistically significantly elevated from one (Tran and Little 2017). These studies used non-cancer and cancer causes of death in their determinations. Based on the work of Tanaka et al. and Zander et al. (see Table 5), animals exposed to LDR lived longer cancer-free than similar mice exposed to the same dose at HDR. Causes of death were similar for control and gamma-exposed animals, although the time to expression of cancer in these animals was more rapid in the gamma-exposed animals than in the controls (Zander et al. 2020). Interestingly, animals sham-irradiated with 120 fractions (i.e., taken to the chamber but not irradiated) had a significant increase in lymphoma incidence over other sham-irradiated animals (i.e., fewer trips to the chamber), and also when compared to non-sham-irradiated animals; this suggests that controls must be carefully considered and any radiation effect may be minimal compared to such environmental factors. Animals exposed to 120 fractions of radiation were not included in this analysis. They had an apparently a lower incidence than the sham-irradiated, but more work is needed to understand this. This study highlights the necessity to have suitable control groups. LDR studies with large numbers of animals were also performed at the IES facility in Aomori Prefecture in Japan. A comparison of males revealed that mice exposed to LDR (0.4 Gy over 400 fractions for 22 h per day, 1.1 mGy/day) had similar causes of death as animals that received high-dose-rate exposures (8 Gy over 400 fractions for 22 h per day, 21 mGy/d) (Tanaka et al. 2007, 2017; Braga-Tanaka et al. 2018a). Female mice, on the other hand, had some dose rate-specific differences noted in the digestive system and circulatory system, which were higher in the animals receiving the higher dose rate than those exposed to a lower dose rate. A comparison of their studies to those by Zander et al. (2020) revealed remarkable similarities in both sexes except in digestive system, respiratory system, and non-neoplastic endpoints. It is possible that differences in ventilation, bedding, and diet could have contributed to these differences.

Studies carried out on flies in parallel above ground (at the reference laboratory at L’Aquila University) and below ground (at the LNGS underground laboratory) have shown that the maintenance in extremely low-radiation environment prolongs the life span, limits the reproductive capacity of both male and female flies, and affects the response to genotoxic stress. These effects were observed as early as after one generation time (10–15 days) and are retained in a trans-generational manner (at least for two more generations) (Morciano et al. 2018a). It is interesting to note that organisms well known to be radioresistant can sense such small changes in the environmental radiation.

Developmental and morphometric endpoints were also investigated in DULs. Data so far obtained on lake whitefish embryos have shown a significant increase in body length and body weight of up to 10% in embryos reared underground, suggesting that incubating embryos inside the SNOLAB can have a subtle yet significant effect on embryonic growth and development (Thome et al. 2017; Pirkkanen et al. 2021). Experiments were also performed using the nematode Caenorhabditis elegans at WIPP have shown that worms growing in the below normal radiation environment had faster rates of larval growth and earlier egg laying; furthermore, more than 100 genes were differentially regulated, compared to normal background radiation levels (Van Voorhies et al. 2020).

Based on these studies, it is clear that at least some examined dose rate effects are evident at the whole organism level.

#### Non-cancer endpoints: inflammation and other systemic effects

The influence of LDR exposures on inflammatory responses was studied using two different animal models: ApoE−/− mice that develop atherosclerosis at a high frequency (Mitchel et al. 2011, 2013; Mathias et al. 2015; Ebrahimian et al. 2018b) and MRL-lpr/lpr mice (Ina and Sakai 2005) that develop a systemic lupus erythematosus-like syndrome. While one can argue that both mouse models have only a moderate relationship to human disease, the effects of radiation exposures particularly at low doses were interesting. In all cases, exposure of animals to LDR radiation exposure demonstrated enhanced life expectancy, in most cases accompanied by either a reduction in pro-inflammatory responses (Mathias et al. 2015) or by an enhanced expression of anti-inflammatory effects (Ebrahimian et al. 2018b). These were evident at lower dose rates but not high-dose rates when they were compared within the study. The protective effects of LDR exposures were not dependent on p53 (Mitchel et al. 2013). Taken together, these results suggest that LDR radiation can inhibit inflammatory responses under the appropriate conditions.

#### Non-cancer endpoints: cataract

Acute exposure to ionising radiation has provided clear evidence of an increased incidence of cataract. However, limited studies have been carried out specifically to address the effect of dose rate on radiation-induced cataract. The most comprehensive study to date (Barnard et al. 2019) exposed C57BL/6 mice to gamma-radiation at 0.84, 3.7, or 18 Gy/h, and found an inverse dose rate response in cataract formation in the lens of the eye. This supports previous epidemiological evidence as reviewed in Hamada et al. (2016).

#### Discussion

In addition to studies described here, there are other non-cancer effects of ionising radiation, particularly cardiovascular disease, that have been well studied using acute radiation exposure. However, specific experiments to establish the effect, if any, of dose rate have yet to be addressed.

Animal research is always dependent on control studies, ensuring that sham-irradiated animals are appropriately tested and that accurately matched controls are being examined. Numerous and extensive studies have documented the impact of the mouse strain on results, since strain-specific differences in pathology (particularly cancer type) and even radiation sensitivity have been noted in the literature (Reinhard et al. 1954; Lindsay et al. 2007). Cross-comparisons of animals from one study to another may be limited by these concerns. In addition, long-term low-dose experiments often require very large animal populations to identify significance of potentially small effects. In addition, LDR studies require not only large numbers of animals but also housing of animals sometimes for years to reach cancer and lifespan endpoints.

Despite these limitations, animal studies have the advantage of examining the total body experience, keeping cells in the context of the tissue, including immune, circulatory, and other systems of the body. This allows for studies on multiple impacts on endpoints and not just single-cell impacts examined in cells in culture. The ability to manipulate specific genes through transgenic mice provides a mechanism by which one can examine the impact of under- or over-expression of these genes. Animal studies also have the advantage (over human epidemiologic work) of having carefully controlled conditions to allow for the best assessment of radiation effects.

#### Conclusions from animal studies

There have been several large-scale animal studies examining dose rate effects. In general, animals exposed to the same dose of radiation at LDR survived longer than those exposed HDR. In addition, the major cause of death in these animals was cancer induction (Tran and Little 2017), although the type of cancer differed in different mouse strains. Studies of inflammatory responses suggest that LDR radiation exposure may inhibit inflammation under appropriate conditions, which, along with an adaptive response, could explain the extended lifespan seen at low-dose rates. Cataract induction (much like results shown for mutations in cellular studies) points to the existence of an inverse dose rate effect.

While radiation exposure has been shown to modulate cancer induction differently in male and female mice (with certain cancers predominating in each sex depending in part on mouse strain), there were few dose rate-specific differences observed in cancer induction between the two sexes. Some non-cancer endpoints, such as digestive system disorders and respiratory disorders, were shown to have sex-specific differences with LDR exposure.

## Dose rate effects in human populations

### Cancer risk epidemiology

To date, most epidemiological studies have focused on risk of cancer after exposure to ionising radiation. These studies of exposure to ionising radiation have included persons who have experienced a wide range of doses received at a wide range of dose rates (McLean et al. 2017; Kamiya et al. 2015). On the one hand, there are the Japanese survivors of the atomic bombings of Hiroshima and Nagasaki and patients treated with radiotherapy, who received a range of doses at an HDR, and on the other hand, there is the general population chronically exposed to a range of LDR of terrestrial gamma and cosmic background radiation. In addition, there are other groups, such as patients undergoing exposure to radiation for medical diagnostic purposes and workers who have experienced a series of low-level exposures in their workplaces.

#### A-bomb survivors

The Japanese atomic-bomb survivors are usually adopted as the reference group for HDR exposures, because the Life Span Study (LSS) cohort has been the subject of careful study and there is little ambiguity in considering a group that has experienced an excess risk of cancer as a result of receiving moderate-to-high doses during a brief exposure to radiation of a few seconds.

More specifically, because of the atomic-bomb explosions over Hiroshima and Nagasaki, radiation exposures of the inhabitants of both cities were due to prompt and delayed radiation, primary and secondary radiation, and gamma and neutron radiation. At a distance of 1000 m from the hypocentre in Hiroshima, for example, the highest contribution to kerma free-in-air (2.77 Gy) was from delayed gamma radiation (gamma radiation produced by the decay of fission products in the rising fireball), which lasted for about 10 s and, consequently, resulted in a dose rate of 0.277 Gy/s. The second highest contribution to kerma free-in-air (1.38 Gy) was from prompt secondary gamma radiation (from prompt neutrons produced during the explosion that resulted in additional gamma radiation when they were transported through the atmosphere and interacted with air and soil), which lasted for about 0.2 s and, consequently, resulted in a dose rate of about 6.9 Gy/s. The third highest contribution to kerma free-in-air (0.24 Gy) was from prompt neutrons (which were produced during the explosion and transported through the atmosphere to the ground), which lasted for only about 10 µs and, consequently, resulted in an HDR of 2.4 × 10^4^ Gy/s. Finally, the fourth highest contribution to kerma free-in-air (0.07 Gy) was from prompt primary gamma radiation (which was produced during the explosion), which lasted for only about 1 µs and, consequently, resulted in a HDR of 7 × 10^4^ Gy/s. Table 6 summarises these dose and dose rate contributions for distances from the hypocentre of 1000 and 2000 m. Similar values for kerma free-in-air hold for Nagasaki. Kerma is calculated here as sum of the kerma from gamma radiation and neutron radiation; for details, see Rühm et al. (2018).

**Table 6:** Dose and dose rate contribution (based on kerma free-in-air) of various radiation sources after the explosion over Hiroshima, at ground ranges (distances from the hypocentre of the explosion) of 1,000 and 2,000 m (Rühm et al. 2018). Similar orders of magnitude hold for exposures due to the explosion over Nagasaki

Radiation source	Estimated duration of exposure	Dose (Gy) at 1,000 m	Resulting dose rate (Gy/s) 1,000 m	Dose (Gy) at 2,000 m	Resulting dose rate (Gy/s) 2,000 m	Resulting dose rate (mGy/h) 2,000 m ^a^
Delayed gamma radiation	10 s	2.77	0.277	0.040	4.0 × 10.^−3^	1.44 × 10.^4^
Prompt secondary gamma radiation	0.2 s	1.38	6.9	0.035	0.17	6.12 × 10.^5^
Prompt neutrons	10 µs	0.24	2.4 × 10.^4^	0.0004	40	1.44 × 10^9^
Prompt primary gamma radiation	1 µs	0.07	7.0 × 10.^4^	0.002	2.0 × 10.^3^	7.20 × 10^9^

^*a*^* Dose rates in mGy/h are also given, to facilitate comparison with data shown in Fig. **1**, although due to the brief nature of the exposure, the rate measure per hour is misleading*

Furthermore, the survivors experienced an exposure that was effectively a uniform whole-body exposure to gamma radiation (although there was a generally small component of exposure to high-LET neutrons that needs to be borne in mind), so that all organs/tissues were exposed at doses that are approximately equal (although smaller for organs/tissues that are deeper within the body).

Finally, a survivor located at 1000 m distance from the hypocentre at time of bombing had experienced a mean dose rate of 2.4 × 10^3^ Gy/s (8.6 × 10^9^ mGy/h) if the dose rates of the four components given in Table 6 were weighted by their corresponding free-in-air kerma values. Similarly, a survivor at 2000 m had experienced a mean dose rate of 5.2 × 10^1^ Gy/s (1.9 × 10^8^ mGy/h). If the contribution from prompt neutrons is multiplied by a factor 10 to account for an increased relative biological effectiveness of neutrons as compared to gamma radiation, these mean dose rates translate to 8.8 × 10^4^ Gy/s (3.2 × 10^11^ mGy/h) and 6.9 × 10^1^ Gy/s (2.5 × 10^8^ mGy/h) at 1000 m and 2000 m, respectively.

#### Medically exposed cohorts

Medical exposures for diagnostic purposes involve doses that are much lower than the (usually localised) doses received during radiotherapy. While the doses received from discrete external exposure radio-imaging procedures are likely to be low, a series of diagnostic exposures, such as computed tomography (CT) scans, could produce cumulative doses that are > 100 mGy (Rehani et al. 2019). It is important to consider that the highest doses may be confined to tissues that are in the vicinity of that part of the body under scrutiny, and the individual exposures could be temporally separated by periods of days. Nonetheless, dose rates during exposure are likely to be moderate-to-high. This potential mix of low dose and HDR effects could lead to difficulties of interpretation, because the two effects described by the two factors, LDEF and DREF, cannot be distinguished.

Considering that the typical tissue dose received during a CT-scan is about 10 mGy and although an examination lasts between 5 and 20 s or so, the vast majority of the dose is delivered as while passing through the ring (under the direct beam), which usually takes less than 1 s. The dose rate is therefore of the order of 10 to 20 mGy/s. Obviously, precautions should be taken as this estimated dose rate is variable depending upon the patient's corpulence, the location of the organ/tissue, and, of course, the scanner settings (current, tube voltage and rotation speed of the X-ray tube, table movement speed, collimation, and filtration) among other considerations.

Studies of those being treated with radiotherapy pose rather more problems of interpretation, because the exposure is generally more localised and being used to treat diseased tissue. This results in high doses to tissues where the radiation is being directed and a gradient of doses to normal tissue away from the focus of treatment, producing a range of doses to healthy tissues being exposed, mainly from scattered radiation.

A classic radiotherapy treatment corresponds to a dose of 2 Gy per fraction (perhaps 20 or more fractions) localised as much as possible to the tumour, with a treatment duration typically around a few minutes depending on the treatment technique. The mean dose rates delivered by the linear accelerators used for radiotherapy treatment are limited to 6 Gy/min and can reach dose rates of up to 24 Gy/min for flattening filter free photon beam. Also, some recent techniques in development of FLASH radiotherapy produce dose rates that are still higher, at mean dose rates in excess > of 40 Gy/s. Flash radiotherapy is based on a series of very short pulses (with a duration of a few microseconds) delivered over a total duration of some milliseconds. Therefore, within one of these pulses, the dose rate can reach extreme values of several 10^5^ Gy/s (Esplen et al. 2020). The competing effects of cell killing at high doses and HDR will depress the risk per unit dose of cancer, which is why comparisons between effects in patients receiving high-level exposure as therapy and those in groups exposed at lower levels need to be conducted with considerable care. A further complication is that the disease being treated with radiotherapy and other therapies in the treatment regimen (chemotherapy, for example) could affect the risk posed by radiation exposure.

Long-term health effects of radiotherapy have been demonstrated for both cancer (Berrington de Gonzalez et al. 2013) and non-cancer diseases, especially diseases of the circulatory system (Little 2016). For lower doses associated with medical exposure, induction of DNA damage by a CT-scan examination has been demonstrated (Jánošíková et al. 2019). Several epidemiological studies investigated the effects of radiation exposure due to CT scans in childhood. Even if the estimated risks are influenced by potential biases and are associated with large uncertainties, accumulated results show that CT exposure in childhood appears to be associated with increased risk of (at least, certain types of) cancer (Abalo et al. 2021). Nevertheless, all these results derived from medical studies relate to radiation exposures at high- or very-high-dose rate.

#### Occupationally and environmentally exposed cohorts

Many studies have been published dealing with exposure of various groups of individuals to low-dose rates of ionising radiation. Among these are occupationally exposed cohorts such as, for example, air crew (Hammer et al. 2014), Western nuclear workers (Leuraud et al. 2015; Richardson et al. 2015), Russian Mayak workers (Sokolnikov et al. 2015, 2017; Kuznetsova et al. 2016), Chernobyl emergency workers (Ivanov et al. 2020a), and others (Shore et al. 2017). Table 7 summarises the typical cumulative doses and dose rates for these cohorts. Groups of individuals exposed to high natural background radiation have also been investigated, especially in Kerala, India (Nair et al. 2009; Jayalekshmi et al. 2021), and Yangjiang, China (Tao et al. 2012), as well as those exposed to man-made contaminations, such as the Techa River population in the Southern Urals of Russia (Krestinina et al. 2013; Davis et al. 2015) and the inhabitants of buildings containing ^60^Co contaminated steel in Taiwan (Hsieh et al. 2017).

**Table 7:** Summarises typical cumulative doses and corresponding dose rates estimated for a number of cohorts (as given in (Rühm et al. 2018)), as compared to cumulative external doses and dose rates for the general population taking into account a total exposure from cosmic radiation and cosmogenic radionuclides of 0.39 mSv annual effective dose (typical range: 0.3 – 1.0 mSv/y) and a total external terrestrial radiation exposure of 0.48 mSv annual effective dose (typical range: 0.3 – 0.6 mSv/y) [taken from Table 31 of Annex B of the UNSCEAR 2000 report (UNSCEAR, 2000)]

Exposed population	Cumulative dose	Corresponding dose rate	Remark	Reference
General population	50 – 130 mSv	0.07–0.2 µSv/h	Calculated from annual effective dose from external radiation sources, for world population; cumulative lifetime doses assume an age of 80 years	(UNSCEAR, 2000)
Air crew	< 200 mSv	2 (< 6) µSv/h	Effective dose, mostly from neutrons and protons (which contribute about 60%-80% of the total effective dose depending on flight altitude, latitude and solar activity); Dose rate estimate based on mean annual effective dose and assumed 900 flight hours per year; cumulative dose assumes 40 years of work	(Frasch et al. 2014) Mares et al. (2009) Bottollier-Depois et al. (2009), (Chen and Mares, 2008)
Nuclear workers	20.9 mGy	0.4 µGy/h	Colon dose; dose rate based on mean reported cumulative dose for those with positive recorded dose, average length of follow-up, and assumed 2,000 working hours per year;	Richardson et al. (2015)
Mayak workers	510 (0 – 6800) mGy^a^	< 150 µGy/h^a^	Personal dose equivalent (H_p_(10)); Dose rate estimated based on annual dose and assumed 2,000 working hours per year	Sokolnikov et al. (2015)
Chernobyl workers	160 mGy	320 µGy/h	Personal dose equivalent (H_p_(10)); first year after the accident; dose rate calculated based on individual time of employment and assumed continuous exposure	Ivanov et al. (2020a)
High radiation background, Kerala, India	161 mGy	< 1 µGy/h	Mean absorbed colon dose; dose rate estimate based on measurement of a randomly selected subset of the cohort	Nair et al. (2009) Jayalekshmi et al. (2021)
Techa population	400 (0–9,000) mGy	External: 4.3 (< 25) μGy/h Internal: 14 (< 340) μGy/h	Red bone marrow dose (from external and internal exposure, where the internal exposure is mainly from Sr-90); dose rates for 1951 assuming chronic exposure	Krestinina et al. (2013) Davis et al. (2015)
Dwellings containing ^60^Co contaminated steel, Taiwan	48 (< 1–2,363) mGy	Average about 0.5 to 1 μGy/h	Dose cumulated between 1982 and early 1990s; mean dose rate estimated from period of habitation	Hsieh et al. (2017)

^*a*^ Only external exposures have been included

Occupational exposures are predominantly received at an LDR, albeit that cumulative doses can be moderate or even high, but consisting of a series of many discreet, small doses received over a working lifetime (Wakeford 2021). Of particular importance are the studies of the workers at the Mayak nuclear complex in Russia and the International Nuclear Workers Study (INWORKS). INWORKS is an international collaborative study of mortality in nuclear workers from the UK, France, and five sites in the USA (Leuraud et al. 2015; Richardson et al. 2015). These are powerful studies involving large numbers of workers, some of whom have accumulated moderate-to-high doses, and the findings of these studies can offer substantial information on dose rate effects when compared with those of the Japanese atomic-bomb survivors.

Shore et al. (2017) made a detailed examination of the excess relative risk (ERR) per unit dose (ERR/Gy) reported by LDR studies (mainly occupational) for solid cancer (all cancers excluding leukaemia, lymphoma, and multiple myeloma) in comparison with the ERR/Gy found in equivalent analyses of the LSS cohort of the Japanese atomic-bomb survivors. Although the results of the Mayak workforce provided support for a DREF of 2, the other occupational studies did not indicate that a reduction in ERR/Gy to account for lower dose rates was required. In particular, the ERR/Gy estimate for INWORKS was compatible with a DREF of 1. When excluding studies with mean doses above 100 mSv (therefore excluding the Mayak worker cohort and the Kerala study), then the estimated DREF was compatible with a value of 1 (Shore et al. 2017).

Recently, Leuraud et al. (2021) made a detailed comparison of the ERR/Gy estimate for solid cancer obtained from INWORKS and from the LSS, selecting subgroups from these studies that were as closely aligned as possible. The ERR/Gy estimates for INWORKS and the LSS were very close, confirming that INWORKS offers little support for any reduction in ERR/Gy for solid cancer derived from the Japanese atomic-bomb survivors when applied to LDR exposures. However, the potential influence of baseline cancer risk factors upon radiation-related risks must be borne in mind when making such comparisons (Wakeford 2021).

Preston et al. (2017) conducted a similar exercise for Mayak workers, comparing the ERR/Gy for mortality from solid cancers excluding lung, liver, and bone cancers (the cancers expected to be associated with plutonium deposition) in the Mayak workforce with that obtained from the LSS cohort members exposed as adults. The ratio of the Mayak and LSS risk estimates pointed to a DREF of 2–3. Similar conclusions were reached by Hoel (2018).

Another recent synthesis considered cancer in epidemiological studies with mean cumulative doses below 100 mGy; therefore excluding, for instance, the Mayak worker cohort and the Kerala natural background radiation cohort (Hauptmann et al. 2020). When focusing on adulthood exposure, the meta-analysis included only LDR studies. The meta-analysis of these studies produced an ERR at 100 mGy of 0.029 (95% CI 0.011 to 0.047) for solid cancers (based on 13 LDR studies) and of 0.16 (95% CI 0.07 to 0.25) for leukaemia (based on 14 LDR studies). The authors concluded that these LDR epidemiological studies directly support excess cancer risks from low doses of ionising radiation, at a level compatible with risk estimates derived from the Japanese atomic-bomb survivors (Hauptmann et al. 2020).

A further strand of evidence on the DREF and DDREF comes from consideration of the findings of studies of those exposed to radiation in the environment. Foremost among these studies are those of residents of areas of high natural background gamma radiation in Yangjiang, China (Tao et al. 2012), and Kerala, India (Nair et al. 2009; Jayalekshmi et al. 2021), and of riverside communities along the Techa River, which was heavily contaminated by radioactive discharges from the Mayak installation in the late-1940s and 1950s (Davis et al. 2015; Krestinina et al. 2013). The Yangjiang and Kerala studies offer little evidence for an excess risk of solid cancer resulting from high natural background gamma radiation. In particular, the latest findings from Kerala (Jayalekshmi et al. 2021) suggest that the ERR/Gy for the incidence of all cancers excluding leukaemia following chronic exposure to LDR gamma radiation may be significantly less than that following acute exposure during the atomic bombings of Japan, although some criticisms have been expressed about the quality of the data used in the Kerala study (Hendry et al. 2009). Also, it is puzzling that the latest analysis of cancer incidence in Kerala (Jayalekshmi et al. 2021) includes 135 cases of leukaemia, but that no quantitative findings for leukaemia are presented.

In contrast, analysis of the Techa River data for mortality (Schonfeld et al. 2013), and incidence of (Davis et al. 2015), solid cancers provides evidence for an excess risk related to enhanced exposure to radiation as a result of radioactive contamination, but the ERR/Gy estimates are similar to those for the LSS and so do not indicate a DREF greater than 1. This conclusion is supported by the study of Preston et al. (2017), comparing solid cancer incidence and mortality in the Techa River and LSS cohorts, which found that ERR/Gy estimates for the two cohorts were very similar for both solid cancer incidence and mortality.

Contamination of construction steel with cobalt-60 in Taiwan in the early 1980s led to several thousand people being exposed at an LDR to elevated levels of gamma radiation over a period of about 10 years (Hsieh et al. 2017). In a cohort of exposed people, indications of excess incidence rates of leukaemia (excluding CLL) and solid cancers (particularly breast and lung cancers) that are related to estimated doses from ^60^Co have been reported (Hsieh et al. 2017), but the precision of risk estimates is insufficient to draw conclusions about an effect of dose rate.

Several large case–control studies have been conducted recently of childhood cancer, in particular childhood leukaemia, in relation to natural background radiation exposure. This interest principally arises because of the prediction of standard leukaemia risk models derived from LSS data that around 15–20% of childhood leukaemia cases in the UK might be caused by background radiation exposure (Wakeford 2004; Wakeford et al. 2009) and that sufficiently large case–control studies should be capable of detecting such an effect (Little et al. 2010). However, the results of large nationwide studies have been mixed and further work is required before reliable conclusions can be drawn (Mazzei-Abba et al. 2020).

A summary of the ERR/Gy estimates reported from main studies of occupational and environmental exposure to radiation at an LDR (and comparisons with the ERR/Gy estimates from the LSS, where available) is provided in Table 8. However, it must be borne in mind that differences in baseline cancer rates in these populations may affect the ERR/Gy estimates, as well as any effect of different dose rates (see discussion further below).

**Table 8:** Estimates of Excess Relative Risk per Gy (ERR/Gy) for studies of solid cancer (or all cancers excluding leukaemia) in cohorts with low-dose-rate (LDR) radiation exposure and corresponding estimates from the Life Span Study (LSS) of atomic-bomb survivors

Study population	Cancer grouping	LDR cohort		Corresponding LSS (exposed adults)	
		ERR/Gy	95% CI	ERR/Gy	95% CI
INWORKS (Leuraud et al. 2021)	All solid cancer mortality	0.29	(0.03, 0.58)	0.28	(0.16, 0.40)
Mayak workers (Preston et al. 2017)	All solid cancers excluding lung, liver and bone cancer, mortality	0.16	(0.07, 0.26)	0.46	(0.18, 0.85)
Techa River residents (Preston et al. 2017)	Solid cancer mortality	0.6	(0.04, 1.3)	0.5	(0.4, 0.6)
Techa River residents (Preston et al. 2017)	Solid cancer incidence	0.8	(0.1, 1.5)	0.6	(0.46, 0.65)
Russian Chernobyl liquidators (Ivanov et al. 2020a) (Ivanov et al. 2020b)	Solid cancer mortality	0.67	(0.2, 1.2)^*^	0.23	(0.12, 0.34)
Russian Chernobyl liquidators (Ivanov et al. 2020a)	Solid cancer incidence	0.48	(0.1, 0.8)^*^		
Kerala, India (adults) (Jayalekshmi et al. 2021)	Incidence of all cancers excluding leukaemia	− 0.05	(− 0.33, 0.29)	0.34	(0.22, 0.45)
Yangjiang, China (adults) (Tao et al. 2012)	Mortality from all cancers excluding leukaemia and liver cancer	0.19	(− 1.87, 3.04)	0.49	(0.35, 0.63)
Taiwan dwellings (all ages) (Shore et al. 2017)	Incidence of solid cancers	0.3	(− 0.4, 0.9)	1.24	(0.96, 1.53)
Meta-analysis of 22 LDR studies (Shore et al. 2017)	Solid cancers (mortality + incidence)	0.15	(0.06, 0.23)	0.45	
Meta-analysis of 16 low-dose (< 100 mSv) LDR studies (Shore et al. 2017)	Solid cancers (mortality)	0.41	(0.12, 0.71)	0.39	
Meta-analysis of 14 low-dose studies (< 100 mSv) (Hauptmann et al. 2020)	Solid cancers (mortality + incidence) after adulthood exposure	0.29	(0.11, 0.47)	0.27/0.64 ^a^	

^*^Approximate confidence intervals; *ERR* excess relative risk, *CI* confidence interval

^a^Males/females

#### Dose rates due to cosmic radiation for astronauts

For completeness and comparison with other situations of human exposure discussed in this review, some typical traits from space exposure are given below. This is despite astronaut exposures being governed by mostly high-LET radiation.

Recently, the radiation dose on the surface of the moon was measured as part of the Chinese Chang’E 4 mission which landed on the moon on 3 January 2019. The mission included the Lunar Lander Neutrons and Dosimetry experiment, which provided a mean dose equivalent rate from galactic cosmic radiation (GCR) of 57.1 ± 10.6 µSv/h. For comparison, at the same time period, the dose equivalent rate onboard the International Space Station (ISS) was 731 µSv/d or about 30 µSv/h when averaging over the contributions from the GCR and from protons in the South Atlantic Anomaly (Zhang et al. 2020a).

As for Mars, data measured by the Mars Science Laboratory during a cruise to Mars indicate dose equivalent rates of about 1.8 mSv/d (75 µSv/h) (Zeitlin et al. 2013), while the Curiosity Rover measured dose equivalent rates of about 0.6 mSv/d (25 µSv/h) on the Mars surface (Hassler et al. 2014). Hence, a total mission to Mars (taking 180 d to Mars, 500 d on Mars, and another 180 d back to Earth) would roughly accumulate 1 Sv (Hassler et al. 2014).

During a large solar particle event, dose rates can be even higher, albeit only during a short period of time. Based on measurements of the Cosmic Ray Telescope for the Effects of Radiation (CRaTER), Schwadron and co-workers estimated the dose rates obtained by astronauts from solar energetic particles (SEPs). For the SEP event that occurred in September 2017, they found that during an extravehicular activity, an astronaut would have received a dose of 170 mGy ± 9 mGy in 3 h (average of 57 mGy/h). Extreme events could result in significantly higher dose rates (Schwadron et al. 2018). This compares to dose rates reported by Dyer et al. who estimated retrospectively that for a hypothetical Concorde flight in 1956 during the event on February 23, dose rates at an altitude of 17 km might have been as high as 0.5 mSv/h, which is about a 100 times higher than those at typical flight altitudes (Dyer et al. 2003).

### Epidemiology for non-cancer endpoints

Evidence for increased risks of incidence and mortality from Diseases of the Circulatory System (DCS) and specific types of DSC (particularly ischaemic heart disease, myocardial infarction, and stroke) were observed in populations exposed to HDR, especially in patients treated with radiation therapy and survivors of atomic bombings at Hiroshima and Nagasaki (Shimizu et al. 2010; Darby et al. 2005; McGale et al. 2011) about 10–20 years ago. ICRP Publication 118 (Stewart et al. 2012) classified DCS as tissue reactions, with a suggested threshold due to acute and fractionated/prolonged exposures of 0.5 Gy (absorbed dose to the brain and blood vessels) for radiological protection purposes. In the last decades, several studies of populations exposed at LDR also demonstrated associations between cumulated dose and DCS risk, in the Mayak worker cohort (Azizova et al. 2018, 2015), in other groups of nuclear workers (Gillies et al. 2017a, b; Zhang et al. 2019; de Vocht et al. 2020), and Chernobyl liquidators (Kashcheev et al. 2016, 2017). However, uncertainties relating to the shape of the dose–response in the low-dose region are considerable, and there are broader issues concerning the interpretation of these epidemiological studies (Wakeford 2019). Up to now, available data do not allow a precise quantification of a potential modifying impact of dose rate on the dose–risk relationships.

Excess risks of posterior subcapsular and cortical lens opacities (cataract) at low-to-moderate doses and dose rates have also been reported in Chernobyl liquidators, US Radiologic Technologists and Russian Mayak nuclear workers (Little et al. 2021). Nevertheless, determination of a potential modifying impact of dose rate on the dose–risk relationship from these data is difficult to assess.

### Discussion

#### Summary of results

At present, the results of epidemiological studies that relate to dose rate effects for human health outcomes following radiation exposure suggest a DREF in a range of 1 to 3. Of the large occupational studies, INWORKS points to no dose rate effect for solid cancer mortality after protracted exposure to LDR in the workplace. Conversely, the Mayak workers cohort provides some evidence of a lower ERR/Gy estimate than directly predicted by the LSS data, by a factor of around 2–3.  The seemingly different conclusions on DREF reached from a comparison of the ERR/Gy estimates derived from the LSS with those from INWORKS and Mayak is an important issue that remains to be resolved (Wakeford 2021). Of environmental exposure studies, the Techa River residents provide some evidence for a raised ERR/Gy estimate for solid cancer (incidence and mortality) that is compatible with the LSS data, but with no indication of a lower ERR/Gy estimate, although the power to reveal a dose rate reduction factor of around 2 is limited. On the other hand, the Kerala study does not indicate a raised risk of solid cancer incidence from chronic exposure to raised levels of natural background gamma radiation, and this finding provides evidence of a lower ERR/Gy estimate for solid cancer than the equivalent estimate derived from the LSS (or that there is no increased risk from these levels of exposure). Interestingly, recent meta-analyses of data restricted to low cumulative doses (mean doses below 100 mSv) led to DREF estimates close to 1.

#### Limitation and advantages of low-dose-rate studies

Clearly, knowledge about the effect of dose rate improved substantially over the last 2 decades, thanks to new published results from populations exposed chronically to radiation. Nevertheless, at low doses, the expected risks are small, and difficult to demonstrate. There are still some limitations of those studies addressing low levels of exposure to radiation: accuracy of dose estimates (particularly when doses have had to be reconstructed from historical data), the quality of some cancer incidence data, lack of control of confounding factors (such as smoking), and for many studies, there is still limited statistical power to assess any dose rate effect. Issues, such as improved dosimetry and better control of confounding, must be addressed if the results of these studies are to be properly interpreted. Nonetheless, the construction of large studies such as INWORKS has notably improved the situation in recent years, and efforts to expand these studies to include more study subjects and extend follow-up will inevitably increase power. A systematic analysis of potential impact of biases (confounding and selection bias, sources of dose errors, loss of follow-up and outcome uncertainty, lack of study power, and model misspecification) concluded that the recent epidemiological results showing increased cancer risk at low doses were not likely to be due to methodological bias (Berrington de Gonzalez et al. 2013; Hauptmann et al. 2020). Differences in the relative biological effectiveness (RBE) of radiation between various exposure situations can also play a role in the observed differences—there is some evidence that low-energy photons (X-rays) are more effective than high-energy photons (gamma rays) at causing DNA damage relevant to stochastic effects (NCRP 2018). Also, it should be underlined that, with a few exceptions (Techa River cohort, Taiwanese contaminated dwellings), most of the available data relate to adulthood exposure, and so data on children are clearly lacking.

#### Excess relative risk versus excess absolute risk models

All results presented above have been obtained using excess relative risk models. Wakeford (2021) points out that not only ERR/Gy should be considered when comparing the results of studies of low-dose rates with those of the Japanese atomic-bomb survivors, but also Excess Absolute Risk per unit dose (EAR/Gy). Comparison of ERR/Gy implicitly assumes that it is valid to compare the proportional increase in risk per unit dose between different populations, which is correct if radiation interacts multiplicatively with those other risk factors that are largely responsible for generating baseline cancer rates, but if the baseline rates in the comparison populations differ and the interaction of radiation with other risks is sub-multiplicative, then the ERR/Gy estimates will differ as a consequence of the difference in the baseline rates. This is reflected in the way excess radiation-related risk is transferred from one population to another—in the ICRP system, for most cancers, a 50/50 mixture of ERR/Gy and EAR/Gy is assumed in the transfer of risk (ICRP 2007; Cléro et al. 2019; Zhang et al. 2020b), which is important when baseline rates differ, as they do between the LSS, Mayak and INWORKS cohorts. Consequently, a difference in ERR/Gy between cohorts may be due to a difference in dose rates to which the members were exposed, but it may also be due to a difference in baseline cancer rates if the interaction between radiation and other risk factors is sub-multiplicative, as is the assumption of ICRP for most types of cancer. Therefore, epidemiological findings in relation to dose rates must be interpreted with substantial caution, and should not depend solely upon comparisons of ERR/Gy when baseline cancer rates differ between the populations under study (Wakeford 2021). This point has also been highlighted in a recent UNSCEAR report, comparing the application of different models to specific exposure situations (UNSCEAR 2020).

### Conclusions from epidemiological studies

At high-dose rates, such as people exposed to radiation from atomic bombs and therapeutic radiation, an increase in cancer incidence is clearly observed, particularly for leukaemia and also for some solid cancers. At low-dose rates, knowledge about cancer risks substantially improved over the last 2 decades. Recent epidemiological studies showed an increased risk of leukaemia and solid cancers, even if risk estimates are associated with large uncertainties (Rühm et al. 2022). A dose–risk relationship is clearly demonstrated for diseases of the circulatory diseases at high doses and high-dose rates. However, there are insufficient data at present to conclude if non-cancer effects are affected by dose rate.

## Conclusions and future needs to understand radiation dose rate effects

### Summary of results and conclusions

The present article presents a comprehensive assessment of radiation dose rate studies to date, including epidemiological studies, and in vitro and in vivo experimental studies.

**Fig. 1:**
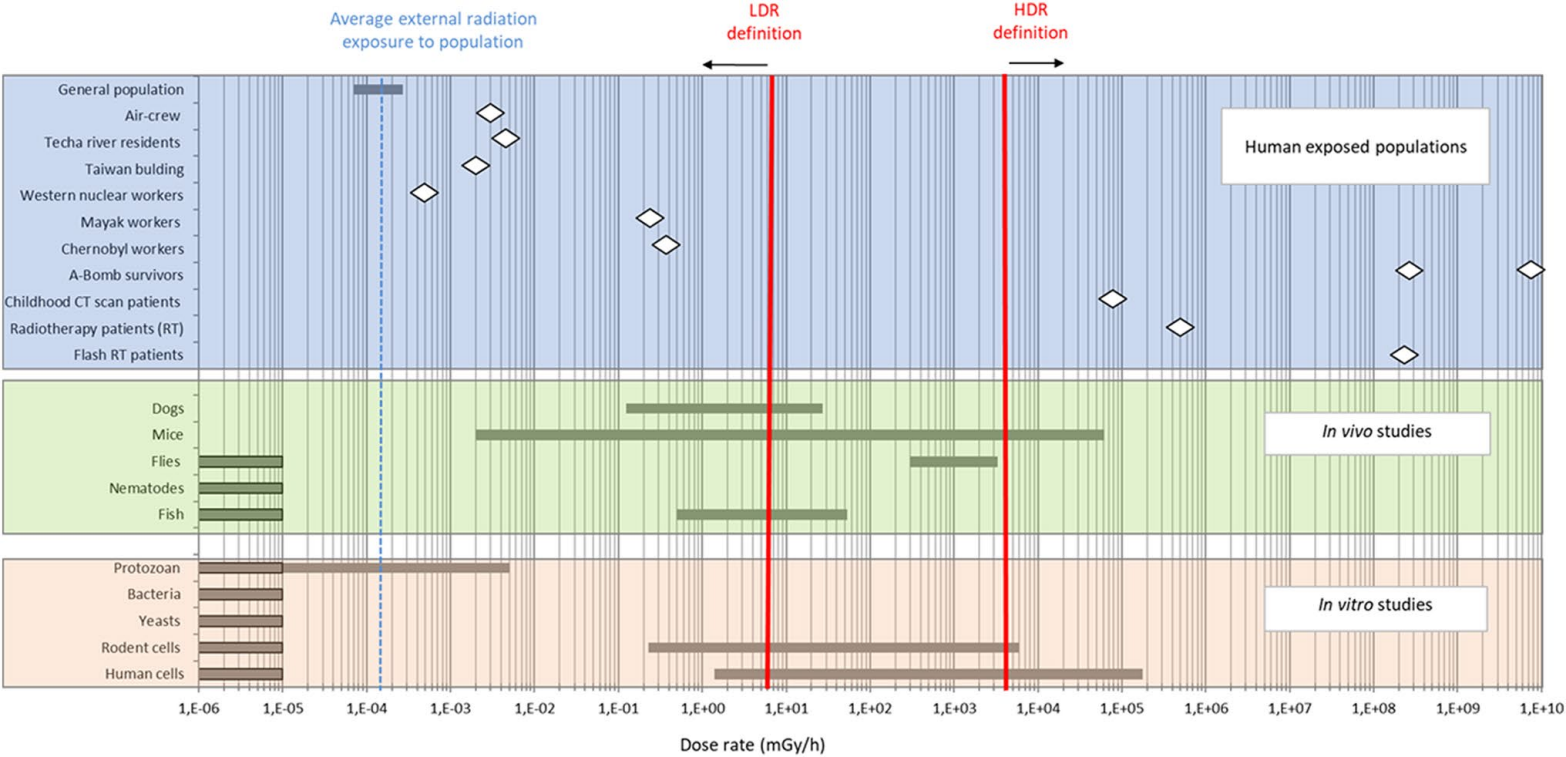
Representation of dose rate ranges (log scale in mGy/h) considered by the different studies presented separately for human, in vivo and in vitro studies. The range of external dose rates received in the general population is shown along with the average 2.4 mGy/h exposure rate worldwide (blue dashed line) (UNSCEAR 2000). LDR definition corresponds to 5 mGy/h and HDR to 0.05 Gy/min (solid red lines). For epidemiological studies, average dose rates in specific situations are shown and represent radiation exposure above background (white diamonds). In comparison, dose rates from the LSS (given in terms of free-in-air kerma) are large and of the order of 1.9 × 10^8^ mGy/h–8.6 × 10^9^ mGy/h, for atomic-bomb survivors located at 2000 m and 1000 m distance from the Hiroshima hypocentre at time of the incident, respectively (for details, see text). For medical exposure situations, average dose rates to the tumours have been considered for radiotherapy and to the area of the body explored for CT-scan. Note that for exposures of atomic-bomb survivors and patients due to diagnostic and therapeutic procedures, times of exposure are short and, therefore, dose rates given in terms of mGy/h may be misleading. For in vivo and in vitro studies, a range is shown that is representative of the dose rates used in selected publications discussed. Data from experiments carried out in Deep Underground Laboratories (DULs) are also reported (grey bars between 1 × 10^–6^ and 1 × 10^–5^ mGy/h)

Figure 1 illustrates the dose rates covered in the studies mentioned in this publication. Note the log scale of dose rates and therefore huge range of dose rates considered.

Of importance is that no in vitro studies have been carried out at the dose rate range corresponding to nuclear worker level or LDR definition. Data from in vitro studies are all performed with dose rates higher than the UNSCEAR LDR definition (0.1 mGy/min or 5 mGy/h) or with extremely low-dose rates for experiments conducted in DUL facilities. This representation also highlights the fact that few data are available outside of epidemiology for dose-rate levels pertinent for radiological protection. At the upper end of the scale, the high-dose rate delivered by nuclear bombing is only partly covered by radiobiology studies. Environmental and occupational human exposures are all around or just above typical background levels, whereas medical exposures are above 1 Gy/h, albeit for short durations. Except for potential astronaut exposure (not included in Fig. 1, for details), there are practically no situations of human exposure in the range around 1–1000 mGy/h. In vivo studies currently have the largest representation of dose rate range.

In conclusion, dose rate effects have indeed been observed. The most compelling evidence comes from:

1. In vivo experiments that generally show reduced inflammation at low-dose rates, unlike higher doses that generate a pro-inflammatory response, particularly at higher dose rates, which usually result in higher total doses. However, it is worth noting that these have typically been seen in models with already increased inflammation.
2. In vivo experiments, very-low-dose rates have generally been shown to increase lifespan through a mechanism thought to be via adaptive response; an inverse dose rate response appears to exist for mutation and cataract formation.
3. In vitro and in vivo experiments carried out below the natural background radiation, where inverse dose rate response has been observed for DNA-related end points (i.e., mutation and DNA/chromosome damage).
4. Certain animal studies demonstrated increased cancer incidence with high-dose rates compared to exposures at low-dose rates when similar total doses were compared particularly when total doses exceeded 0.5 Gy.
5. Changes have been observed in chromosome damage and gene expression with increased dose rates; however, these studies vary in their conclusions and are often difficult to distinguish dose rate from total dose effects.
6. Epidemiological studies, which show no or little reduction of the dose–risk relationship at LDR compared to high-dose rates for cancers (results compatible with an absence of reduction or a reduction by a factor of about 2). There are currently insufficient data to conclude about an effect of dose rate on non-cancer risks.

### Perspectives and recommendations

#### Considerations and requirements for in vitro and in vivo experiments to determine dose rate effects

Based on our experience and review of the literature, it was possible to highlight some recommendations for conducting dose rate experiments to provide informative data (Table 9).

**Table 9:** Recommendations for cell culture and animal experiments for dose rate analysis

Consideration	Recommendation
1.Cell/tissue/species type	Conditions appropriate for experiment and measured endpoint are essential. Investigators need to recognise the importance of experimental systems and endpoints that are adequate for investigating the question
a.Cell line or primary cell or b.Animal strain/model	Use normal primary cells for assessing normal tissue response; consider cell/tissue-specific response; use appropriate genetic alteration
c.Age/sex of donor/organism	Use a variety, including multiple donors, to confirm consistency of results (or determine factors that could lead to result in higher response)
2.Cell culture conditions	Culture conditions that are consistent and relevant to physiological conditions appropriate for the endpoints
a.Confluence	In vivo, cells will usually be confluent with low proliferation rates. Awareness of the effects of culturing cells is essential
b.Proliferation	
c.Oxygen concentration	Use normoxic conditions for very low doses
d.Routine	Maintain consistency (e.g., timing of harvesting/media changes)
3.Animal experiments	Ensure the model chosen is appropriate to the endpoint being investigated
a.Experimental design	Include randomised, appropriate controls that are matched for exposure conditions to irradiated samples. Sham-irradiated controls are needed for all animal experiments
b.Housing conditions	Consider best practice for animal welfare, plus variability introduced via cage mates, enrichment, light, noise, etc.
c.Other stress/variability	Reduce sources of stress (e.g., handing and transport of experimental animals)
4.Ionising radiation exposure	Ensure consistency and appropriate controls
a.Dose rate	Use multiple dose rates to observe dose rate effects, including physiologically relevant LDR
b.Cumulative dose	Include controls to distinguish dose-rate effect from total cumulative dose received
c.Radiation type	Keep exposure type the same to be able to compare between studies
5.Readout/endpoint	Select appropriately and use a variety to confirm effects
6.Molecular (‘omics’, genomic instability, chromosome aberration, histone modifications)	Validate molecular changes using appropriate functional assay
7.Cancer (mutation/unrepaired DNA damage, inflammation, cell death)	Consider sensitivity of assay, suitability to answer question, other contributing factors
8.Non-cancer (inflammation, senescence, altered proliferation, epigenetic age)	

One of the most important considerations in setting up in vitro experiments is the selection of appropriate cells. Although certain cell lines have been used in radiation research for decades and have advantages such as unlimited supply, they often have features that are not found in normal human tissues. For example, many cell lines have lost p53, a transcription factor central to DNA damage response, or do not have normal cell cycle checkpoints, and, therefore, lack relevance when attempting to elucidate a normal tissue response to ionising radiation. As a result, we recommend the use of primary human cells where possible. Although this brings its own set of challenges, including differences between donors, it also allows for factors including the age and sex of an individual to be considered in the assessment of dose rate effect. This is vitally important for identifying populations that may be of increased or reduced risk for radiation effects and necessitates experiments being carried out in cells isolated from multiple donors of varied background to observe any differences. As well as identifying potential ‘at risk’ individuals, this approach will also give more certainty to results seen to be consistent among all donors. To observe a difference, which may only be small, it is important to control for confounding factors, including the cell state and the karyotype. This means that one must take great care to ensure that cells are always at the same level of confluence and proliferation (if possible, in a state that reflects normal physiological conditions). This can be aided by maintaining a consistent routine for cell culture and harvesting samples. Since the level of damage inflicted on cells from LDR radiation may be minimal compared to the damage received from endogenous sources, any small effects from very low doses may be masked by culturing cells under standard culture conditions (21% oxygen) due to higher levels of reactive oxygen species (ROS) present. Within the body, normoxic conditions are around 3–5% oxygen depending upon tissue. Therefore, reducing the oxygen concentration may be necessary to observe very small LDR effects via in vitro experiments. Controlling the karyotype is also very important when analysing the induction of DNA damage. Indeed, deviations from normal karyotype may result in variations in DNA contents impacting energy deposition among DNA.

Another important consideration links the experimental endpoint and selection of cell type. It is known that there is great variation in the responses of different cell types to many stimuli, including ionising radiation. Therefore, it is important to select a cell type that reflects the output being investigated and that any results are not extrapolated to other cell types without empirical evidence being collected for this cell type. To strengthen conclusions going forward, it is important that any results from untargeted molecular approaches, such as genomic or proteomic analysis, are validated by functional assays before accepting any conclusions from the data.

Similar considerations must also be in place for selection of appropriate animal models. Although there is place for genetically altered and inbred strains, typically a more wild-type model will allow for a more normal response and therefore place slightly fewer restrictions on the ability to generalise the results to a wider context. For mouse experiments, an ideal setup would be to test a hypothesis on multiple strains of both sexes and a range of ages to confirm reliability and repeatability of the results. Clearly, consideration must be given to the improvement of the hypothesis being tested and balanced against the number of animals being used.

As the flat and relative homogeneous nature of cell culture is a limitation of in vitro experiments, consideration should be given to more complex cultures, such as organoids, which have a more physiological response and have been developed over the past years to be increasingly sophisticated but also easy to handle.

Finally, the radiation exposure conditions must be considered—type, duration, dose rate, and total dose of radiation. Although all types of radiation are important, in this review, we have focused on low-LET and external exposures. Due to the different type of damage received by individual cells, comparing different qualities of radiation may not be possible and may reflect some of the conflicting conclusions in the current literature. Therefore, we recommend comparing only similar exposures when drawing conclusions on dose rate effect. Conclusions on dose rate effects also require multiple different dose rates to be used to observe trends. This introduces additional challenges such as varied cumulative dose and/or time in culture and therefore cell state. Ideally, cells should receive the same cumulative dose and multiple controls will be required to ensure that any results seen are not due to confounding effects in cell state. For example, harvesting controls at different confluence to reflect the irradiated cultures, or to investigate multiple times post-irradiation to account for the repair of radiation-induced damage. Most previous studies have used relatively high-dose rates (see Table 9), potentially due to the difficulty of seeing effects over confounding factors. To establish any LDR effects going forward, in vitro experiments must include lower dose rates. By taking care to account for other factors as described, this should provide reliable data to allow for conclusion on dose rate effect—whether positive or negative.

Although most of these points could be applied to any in vitro or in vivo experiment, they are of even higher concern in the area of LDR research, where current evidence is at times contradictory and experiments require a high level of accuracy to produce a consistent and reliable outcome. Ultimately, the use of appropriate readouts, extensive use of controls, and consistency will be key in cellular studies to conclusively determine potential dose rate effects from ionising radiation.

From the 2000s until now, much work has been done in the field of low-dose radiation biology that requires more study in animal systems: the roles of different genetic backgrounds and modification of specific genes has been shown to have a striking effect on the radiation response. New modulators of radiation responses include epigenetic effects, such as methylation and histone modification, in addition to expression of non-mRNA RNAs, such as long non-coding RNAs (lncRNAs) and miRNAs. Cytoplasmic effects of low doses (such as gap junctions and mitochondria) have been shown to be important. In many organ systems, stem cells have also provided an impetus for new studies and new hypotheses.

With the development of new technologies (single-cell sequencing, genome-wide sequencing, and others), it is possible to gain much more information about the effects of radiation exposure (and in particular LDR exposures) from single-cell systems, examining the influence of microenvironment and cellular milieu on the endpoint in question. New radiation possibilities have been introduced with specialised facilities that are capable of very low doses and very LDR. As we understand processes in cells differently than in the past, it is possible to probe these new functionalities for the impact of low-dose and low-dose rate exposures.

To progress the area of dose rate research, systematic experimental studies must be designed in coordination with relevant in vivo and in vitro model systems exploiting available facilities. From the comparison between the human exposure scenarios and the currently available in vivo and in vitro data (Fig. 1), it appears that there are dose rate ranges that still need to be investigated. This is now possible taking advantage of the existing irradiation infrastructures. LDR facilities allow the exposure of a range of samples, from cells to organisms, to external gamma irradiation. Moreover, above-ground studies at increasing dose rate can be complemented by experiments carried out in DULs. For radiological protection purposes, it would be extremely relevant to get data on the same organism(s) from a systematic investigation and comparison of doses and dose rates covering sub-background, background, and enhanced doses.

#### Considerations and requirements for epidemiological studies to determine dose rate effects

Epidemiological studies with long-term follow-up are ongoing among populations with either HDR (A-bomb survivors, patients treated with radiation therapy, patients who benefited from medical imaging) or LDR exposure (nuclear workers, populations with environmental radiation exposure). As these studies recruit more participants, have longer follow-up and allow good-quality registration of disease occurrence, more data will be generated which will provide stronger statistical weight to findings on the effects of dose rate.

Potential improvement includes better assessment of dose rate patterns and use of elaborate modelling approaches to better analyse the impact of dose rate on the estimated dose–risk relationship. The use of real-time dose monitoring may help in improving this issue, especially in occupational exposure situations for which radiological protection is continually being improved. Situations of high environmental radiation exposure may also prove to be a good setting for investigating the effects of LDR in the future, if individual dose assessment improves. Within this, specific attention should be given to exposure during pregnancy and during childhood.

As epidemiological data accumulates, we should be able to better estimate DDREF, DREF, and LDEF, along with EAR and ERR differences with dose rate. Across the field of dose rate research, consideration must be given to dose rate effects from different radiation qualities, particularly high-LET sources, such as alpha particles. Also, dose rates at the high extreme, such as flash radiotherapy, should be investigated.

### Future directions

After considering the current understanding of biological effects of dose rate, we conclude by summarising the key points that should be the centre of focus for future research in the area. First, cellular models must be extremely well planned to take into consideration the points highlighted in Table 9, and also using lower dose rates that represent environmentally relevant dose rates. Animal models should consider a range of species and give special attention to variation in effect due to genetic background and age, including embryonic development that thus far appears to be dose rate-dependent. The future addition of existing large databases from large-scale animal studies in Russia, Canada, Japan, and Korea to the developed US and EU databases would of significant benefit to the field.

The focus of radiation research has been on cancer; however, evidence so far suggests that lower dose rates have significant effects on non-cancer effects, including inflammation, which has the potential to be either harmful or beneficial. Uncertainty over the relative contribution of dose rate effects to such endpoints as cardiovascular disease, central nervous system disorders, cataracts, and the corresponding mechanisms responsible require further delineation. These non-cancer endpoints must be prioritised going forward.

Continued work on DDREF, DREF, and LDEF with larger datasets from animals would be of value as they may point to considerations for humans. DDREF applies to lifetime risk of cancer in humans in the calculation of radiation detriment. It would be of interest that a specific attention is given to cancer occurrence in animal experiments, to provide more directly comparable endpoints, since this remains a major uncertainty for human epidemiological work. As noted in this paper, there is huge value to having results from carefully controlled studies for comparison.

Regarding epidemiology, conducting good-quality studies in populations with different radiation exposure patterns is key to increasing our knowledge of the effects of dose rate on health risks. Many studies are underway, and the inclusion of new participants, the improvement of dose reconstruction, and the extension of follow-up duration will be key to improving the interpretation of results. In addition, the initiation of studies on populations with specific exposure profiles (in utero and childhood exposures, repeated medical exposures such as diagnostic imaging or interventional procedures, new radiotherapy techniques) will be very useful. Finally, integration of epidemiological and radiobiological approaches, through the collection of biological material in the design of studies, will be essential to improve our understanding of the effect of dose rate in the future.

Finally, all these separate aspects must be brought together in a concerted effort to better characterise the role of dose rate at the molecular, cellular, organism, and population levels.

Integrating epidemiology and radiobiology approaches will be essential to make strong conclusions on the effect of dose rate; they must complement each other. As so well described by Morgan and Bair (2013): *“Radiation biology research can provide a mechanistic understanding of the effects of low-dose radiation in cells, tissues, organs and organisms. Many of the research tools and technologies to address these issues that we could only dream about in the past are now available and continue to evolve. It is anticipated that using these we can provide a scientific basis that combined with epidemiological studies, chemistry and physics will generate sufficient knowledge that can lead to a rational radiological protection policy to realistically accomplish the objective of maintaining the risks associated with ionising radiation exposures to ‘As Low As Reasonably Achievable’ (ALARA).”*
